# Osa‐miR398b boosts H_2_O_2_ production and rice blast disease‐resistance via multiple superoxide dismutases

**DOI:** 10.1111/nph.15678

**Published:** 2019-02-06

**Authors:** Yan Li, Xiao‐Long Cao, Yong Zhu, Xue‐Mei Yang, Kai‐Ni Zhang, Zhi‐Yuan Xiao, He Wang, Jing‐Hao Zhao, Ling‐Li Zhang, Guo‐Bang Li, Ya‐Ping Zheng, Jing Fan, Jing Wang, Xiao‐Qiong Chen, Xian‐Jun Wu, Ji‐Qun Zhao, Oliver Xiaoou Dong, Xue‐Wei Chen, Mawsheng Chern, Wen‐Ming Wang

**Affiliations:** ^1^ Rice Research Institute and Key Lab for Major Crop Diseases Sichuan Agricultural University Chengdu 611131 China; ^2^ Collaborative Innovation Center for Hybrid Rice in Yangtze River Basin Sichuan Agricultural University Chengdu 611131 China; ^3^ Department of Plant Pathology University of California Davis Davis CA 95616 USA

**Keywords:** *Cu/Zn‐Superoxidase Dismutase*, enzyme activity, H_2_O_2_, *Magnaporthe oryzae*, miR398b, rice resistance, *Superoxide Dismutase*

## Abstract

- miRNAs contribute to plant resistance against pathogens. Previously, we found that the function of miR398b in immunity in rice differs from that in Arabidopsis. However, the underlying mechanisms are unclear.
- In this study, we characterized the mutants of miR398b target genes and demonstrated that multiple superoxide dismutase genes contribute to miR398b‐regulated rice immunity against the blast fungus *Magnaporthe oryzae*.
- Out of the four target genes of miR398b, mutations in *Cu/Zn‐Superoxidase Dismutase1* (*CSD1*), *CSD2* and *Os11g09780* (*Superoxide DismutaseX*,*SODX*) led to enhanced resistance to *M. oryzae* and increased hydrogen peroxide (H_2_O_2_) accumulation. By contrast, mutations in *Copper Chaperone for Superoxide Dismutase* (*CCSD*) resulted in enhanced susceptibility. Biochemical studies revealed that *csd1*,* csd2* and *sodx* displayed altered expression of CSDs and other superoxide dismutase (SOD) family members, leading to increased total SOD enzyme activity that positively contributed to higher H_2_O_2_ production. By contrast, the *ccsd* mutant showed CSD protein deletion, resulting in decreased CSD and total SOD enzyme activity.
- Our results demonstrate the roles of different SODs in miR398b‐regulated resistance to rice blast disease, and uncover an integrative regulatory network in which miR398b boosts total SOD activity to upregulate H_2_O_2_ concentration and thereby improve disease resistance.

## Introduction

In plants, production of reactive oxygen species (ROS), such as superoxide radicals (O^•^_2_^−^), hydroxyl radicals (OH^·^) and hydrogen peroxide (H_2_O_2_), are considered important defense reactions in response to biotic and abiotic stress (Jones & Dangl, 2006; Boller & He, 2009; Saxena *et al*., 2016). The synthesis and homeostasis of ROS in plants are regulated by a series of enzymes, such as the NADPH oxidases (also known as respiratory burst oxidase homologs (Rbohs)), Superoxidase Dismutase (SOD), catalase (CAT) and ascorbate peroxidase (APX). Rbohs are key enzymes in generation of O^•^_2_^−^ by catalyzing the transfer of electrons from NADPH to oxygen (O_2_) (Kaur *et al*., 2014). In Arabidopsis, both *RbohD* and *RbohF* are required for ROS production, as *atrbohD* and *atrbohF* mutants show lower ROS concentrations during incompatible interactions with bacterial and oomycete pathogens (Torres *et al*., 2002). In rice, *RbohD* and *RbohF* are induced by oxidative stress, leading to increased intracellular ROS accumulation (Jang *et al*., 2012). *RbohB* knockdown rice plants display enhanced susceptibility to rice blast fungus *Magnaporthe oryzae*, suggesting that RbohB is required for resistance to rice blast (Nagano *et al*., 2016). SODs act as H_2_O_2_ synthetases, catalyzing the reduction of O^•^_2_^−^ into H_2_O_2_ (Fridovich, 1995; Gill & Tuteja, 2010). SOD isoenzymes are classified into three types according to their affinity for specific metal ions in plants: the Cu/Zn‐Superoxidase Dismutase (SOD) (CSD), the manganese SOD (SOD‐Mn) and the iron SOD (SOD‐Fe) (Mittler, 2002). Overexpression of SOD‐Mn increases plant tolerance against freezing, water deficit, low temperature (McKersie *et al*., 1993, 1996, 1999) and methyl viologen‐induced oxidative stress (Bowler *et al*., 1991; Slooten *et al*., 1995). Overexpression of SOD‐Fe results in enhanced tolerance against methyl viologen in tobacco (Van Camp *et al*., 1996) and maize (Van Breusegem *et al*., 1999). Overexpression of a pea homolog of Arabidopsis CSD2 in tobacco leads to increased tolerance against high light and low‐temperature stresses (Gupta *et al*., 1993a,b). Overexpression of another pea CSD in tobacco enhances ozone tolerance (Pitcher & Zilinskas, 1996). Overexpression of a CSD in rice results in increased resistance to NaHCO_3_ and water stress (Guan *et al*., 2017). Therefore, SODs act as key defense factors against versatile stresses by regulating the H_2_O_2_ concentration in plants. H_2_O_2_ is a vital ROS in biological processes leading to tolerance against various stresses (Kaur *et al*., 2016; Saxena *et al*., 2016).Consistent with the role of ROS in rice immunity, the resistant rice cultivar IRBLKm‐Ts displays higher H_2_O_2_ concentrations than the susceptible cultivar Lijiang XinTuan Hegu (LTH) upon *M. oryzae* infection (Li *et al*., 2014). In addition, overexpression of l‐ascorbate oxidase (AO) enhanced AO‐mediated H_2_O_2_ accumulation, thereby improving resistance to rice stripe virus (Wu *et al*., 2017). ROS homeostasis can be controlled by CAT, a peroxisome‐located H_2_O_2_ scavenging enzyme which catalyzes the reduction of H_2_O_2_ into H_2_O and O_2_ (Racchi, 2013). In addition, APX and glutathione peroxidase are involved in the removal of H_2_O_2_ (Racchi, 2013). Therefore, ROS‐mediated stress tolerance in plants is regulated by the ROS‐homeostasis‐related enzyme system, which, in turn, seems to be fine‐tuned by certain microRNAs (miRNAs) (Baldrich & San Segundo, 2016).

The miRNAs are a category of 20–24‐nucleotide (nt) noncoding RNAs that play important roles in plant development and defense responses by negatively regulating target gene expression (Padmanabhan *et al*., 2009; Katiyar‐Agarwal & Jin, 2010; Baldrich & San Segundo, 2016; Tang & Chu, 2017). miR398 is a conserved miRNA family that suppresses the expression of the SOD family members in many plants, including Arabidopsis (Guan *et al*., 2013; Lu *et al*., 2013), *Brassica rapa* (Yu *et al*., 2012), *Helianthus annuus* (Khaksefidi *et al*., 2015), wheat (Xin *et al*., 2010), switchgrass (Hivrale *et al*., 2016) and Populus (Chen L. *et al.*, 2012; Chen M. *et al.*, 2012). In Arabidopsis, miR398 regulates oxidative stress response by silencing the expression of *CSD1* and *CSD2* (Sunkar *et al*., 2006). In wheat, miR398 and its target genes are responsive to multiple stimuli including cold, wounding and salt stresses (Wang *et al*., 2014). In tomato, miR398a is involved in response to drought stress, and its amounts in sensitive and tolerant genotypes were inversely correlated (Candar‐Cakir *et al*., 2016). In pea, miR398 accumulation is decreased whereas CSD1 expression is enhanced upon water deficit (Jovanovic *et al*., 2014). MiR398 and CSDs also regulate plant disease resistance against pathogens. In Arabidopsis, miR398 overexpression lines display enhanced susceptibility to *Pseudomonas syringae* DC3000 by downregulating *CSD1* and *CSD2* (Li *et al*., 2010a). In barley, *Mla* and *Rom1* negatively regulate the miR398 amount, promoting SOD1 accumulation and enhancing resistance against powdery mildew (Xu *et al*., 2014). In rice, however, miR398b overexpressing lines (*OX398b*) show enhanced basal defenses against *M. oryzae* associated with reduced mRNA amounts of *CSD1*,* CSD2*,* SODX* and *CCSD* (Li *et al*., 2014), indicating that the miR398‐SOD module may play reverse roles in regulation of resistance against pathogens in rice compared to that in Arabidopsis or in barley.

In rice, the SOD gene family contains 15 members, including eight annotated *SOD* genes and seven related genes including the chaperone *CCSD* (Nath *et al*., 2014). Among the eight annotated SOD genes, four of them encode CSDs, including *Os03g22810* (CSD1), *Os07g46990* (CSD2), *Os03g11960* (CSD3) and *Os08g44770* (CSD4). *Os04g48410* encodes a copper chaperone for superoxide dismutase (CCSD) that delivers copper to superoxide dismutase, and *Os05g25850*,* Os06g02500* and *Os06g05110* encode SOD‐Mn, SOD1‐Fe and SOD2‐Fe, respectively (http://rice.plantbiology.msu.edu). Three of the SOD family members, including CSD1, CSD2 and CCSD, were identified as target genes of miR398b by degradome‐sequencing assays (Wu *et al*., 2009; Li *et al*., 2010b). In addition, one uncharacterized gene, *Os11g09780* (designated *SODX* hereafter), is identified as a member of the SOD gene family (Nath *et al*., 2014) and is predicted to be a miR398b target in rice (Wu *et al*., 2009). To address why miR398b positively regulates rice immunity against the blast fungus, we analyzed the function of miR398b's target genes in rice blast disease‐resistance by generating mutants and transgenic lines expressing target mimicry of miR398b (MIM398). Our data indicate that *CSD1*,* CSD2* and *SODX* negatively regulate, whereas CCSD is required for, miR398b‐boosted H_2_O_2_ production, leading to resistance to rice blast disease. H_2_O_2_ concentration seems to be correlated with total SOD enzyme activity upon pathogen infection, which in turn is regulated by the target genes of miR398b. Mutation of CCSD led to decreased CSD and total SOD enzyme activity upon *M. oryzae* infection. However, mutation of CSD1 and CSD2 resulted in increased total SOD enzyme activity, and mutation of SODX led to constitutively higher SOD enzyme activity. Therefore, the target genes of miR398b have antagonistic roles in the SOD enzyme system. Our data demonstrate that miR398b boosts H_2_O_2_ production via multiple SODs, and explain why the function of miR398b in immunity in rice is different from that in Arabidopsis and barley.

## Materials and Methods

### Plant materials and growth conditions

The rice (*Oryza sativa*) *indica* accession Kasalath, *japonica* accessions Nipponbare (NPB) and Taipei 309 (TP309) were used for transgenic analysis. For blast‐resistance and resistant response assays, the wild‐type (WT) control and transgenic lines were grown in a greenhouse at 28 ± 2°C and 70% relative humidity under 12 h : 12 h, light : dark cycles.

### Plasmid construction and genetic transformation

The miR398b‐insensitive versions of *Cu/Zn‐Superoxidase Dismutase2* (*CSD2*_*m*_) was amplified from NPB cDNA with primers CSD2‐*Kpn*I‐F and CSD2‐*Spe*I‐R (Supporting Information Table S1). The miR398b‐insensitive version of *Copper Chaperone for Superoxidase Dismutase* (*CCSD*)*m* was generated by site‐directed mutagenesis using the primers CCSD‐*Kpn*I‐F, CCSDm‐R, CCSDm‐*F*, CCSD‐*Spe*I‐R (Table S1). The PCR fragments were fused to enhanced‐green fluorescent protein (GFP) and cloned into the *Kpn*I–*Spe*I site of the binary vector 35S‐pCAMBIA1300 resulting in the overexpression vector. To construct MIM398, a 331‐bp fragment including the endogenous target mimicry sequences of miR398b (chr5:6397583..6397606) (Wu *et al*., 2013) was amplified from NPB genomic DNA with primers MIM398‐*Kpn*I‐F and MIM398‐*Spe*I‐R (Table S1), and cloned into the *Kpn*I–*Spe*I site of the binary vector 35S‐pCAMBIA1300. *Agrobacterium* strain *EHA105* was selected for rice genetic transformation. Hygromycin B was used for screening the genotype of transgenic plants by means of hygromycin resistance analysis.

### The CRISPR/Cas9 plasmids construct and mutant screen

The CRISPR (clustered regularly interspaced short palindromic repeats)/Cas9 plasmids of target genes were constructed as reported previously (Li *et al*., 2017b). The guide RNA sequences listed in Table S2 were screened by Cas‐OFFinder system (http://www.rgenome.net/cas-offinder/) to avoid potential off‐target‐sites with the screen parameters to allow <3 bp mismatches and one DNA/RNA bulge. The maize ubiquitin promoter (UBI) upstream of a codon optimized hSpCas9(Cong *et al*., 2013) was inserted into binary vector pCAMBIA1300 with hygromycin selection (via hygromycin B phosphotransferase). The original *Bsa*I site present in the pCAMBIA1300 backbone was removed using a point mutation kit (Transgen, Beijing, China). A fragment containing an *OsU6* promoter(Feng *et al*., 2013) and a negative selection marker gene *ccdB* flanked by two *Bsa*I sites and a sgRNA derived from pX260(Cong *et al*., 2013) was inserted into this vector using In‐fusion cloning kit (Takara, Tokyo, Japan) to produce the CRISPR/Cas9 binary vector pBGK032. *Escherichia coli* strain DB3.1 was used for maintaining this binary vector. The 23 bp targeting sequences (including PAM) were selected within the target genes and their targeting specificity was confirmed using a Blast search against the rice genome (https://blast.ncbi.nlm.nih.gov/Blast.cgi) (Hsu *et al*., 2013). The designed targeting sequences were synthesized and annealed to form the oligo adaptors. Vector pBGK032 was digested by *Bsa*I and purified using a DNA purification kit (Tiangen, Beijing, China). A ligation reaction (10 μL) containing 10 ng of the digested pBGK032 vector and 0.05 mM oligo adaptor was carried out and directly transformed to *E. coli* competent cells to produce CRISPR/Cas9 plasmids. *Agrobacterium* strain *EHA105* was selected for rice genetic transformation.

Genomic DNA was extracted from T_2_ transgenic lines and the primers flanking the designed target site (Table S2) were used for PCR amplification. The PCR products (300–500 bp) were sequenced directly and blasted to the WT genome sequence to identify the mutation sites.

### Agrobacterium‐mediated transient expression assay in *Nicotiana benthamiana*

YFP detection and accumulation was assayed as reported previously (Li *et al*., 2017a,b). In order to generate miR398 target‐site reporter fusions, we fused yellow fluorescent protein (YFP) with either the target site of *Superoxide DismutaseX* (*SODX*) at its N‐terminus (*35S:SODX*_*ts*_ *‐YFP*) or with a mutated target site (*35S:SODX*_*mts*_ *‐YFP*) that could not be recognized by miR398b. The sequences of SODX_ts_ and SODX_mts_ were synthesized by annealing gene‐specific primers *SODX*_ts_‐F/R and *SODX*_mts_‐F/R, respectively (Table S1). The isolated fragments were then fused to the N‐terminus of YFP and inserted into *Kpn*I‐*Spe*I sites of binary vector 35S‐pCAMBIA1300. Agrobacterium strain GV3101 was used for agroinfection assay in *N. benthamiana*. In brief, Agrobacterium strain GV3101 harboring the respective expression constructs (*35S:SODX*_*ts*_ *‐YFP*,* 35S:miR398b*,* 35S:MIM398*) was incubated at 28°C overnight in LB media containing kanamycin (50 mg ml^−1^) and carbenicillin (50 mg ml^−1^) on a table shaking at 250 rpm. The bacteria were collected at 800 g for 5 min and resuspended in an MMA buffer (10 mM MES, 10 mM MgCl_2_, 100 mM AS). The Agrobacteria harboring the expression constructs were infiltrated into leaves of *N. benthamiana* for transient expression assay. Leaves were examined at 48 h post‐infiltration (hpi) for image acquisition using a NikonA1 Confocal Laser Scanning Microscope (Nikon Instruments Inc., Shanghai, China). Western blotting analyses were performed to determine the accumulation of YFP. In brief, 15 μg of total extracted protein was electrophoresed on a 10% SDS‐PAGE gel, and then transferred to a membrane. The protein blot was reacted with 3000‐fold‐diluted anti‐GFP sera (Sangon Biotech, Shanghai, D110008, China) and 4000‐fold‐diluted anti‐actin sera (Sangon Biotech, Shanghai, D110007, China), respectively, to detect YFP and actin accumulation.

### Pathogen infection and microscopy

*Magnaporthe oryzae* strains stocked in the laboratory (Guy11, GZ8 and 089) were used in this study. *Magnaporthe oryzae* strains were incubated on Complete Medium at 28°C with 12 h : 12 h, light : dark cycles for sporulation. After 2 wk, the spores (1 × 10^5^ spores mL^−1^) were collected for punch‐ and spray‐inoculation. The lesions in infected leaves were observed at 5 d post‐inoculation (dpi) and the fungal biomass was measured as reported previously (Park *et al*., 2012). In brief, relative fungal biomass was calculated using the DNA amount of *M. oryzae Pot2* against the rice genomic ubiquitin DNA amount by quantitative reverse transcription polymerase chain reaction (qRT‐PCR). For observing the infection process of *M. oryzae*, we inoculated the strain GZ8 on 5‐cm‐long leaf sheaths as described previously (Kankanala *et al*., 2007). The inoculated epidermal layer was excised and analyzed by fluorescence microscopy (Zeiss Axio Imager A2, Carl Zeiss Co. Ltd, Chengdu, China) during 24–48 hpi.

### H_2_O_2_ and O·_2_^−^ measurement

Hydrogen peroxide (H_2_O_2_) accumulations and cell death in infected leaves were observed with the protocol given in Methods S1. The quantification of H_2_O_2_ followed the method described in (Wu *et al*., 2017). Briefly, 50 mg of inoculated fresh leaves were collected and ground with liquid nitrogen. Five hundred microlitres of sodium phosphate buffer (50 mM, pH 7.4) was added to extract H_2_O_2_ and the mixture was centrifuged at 12 000 ***g*** for 20 min at 4°C to pellet cell debris. The supernatant was used for H_2_O_2_ concentration assay with the Amplex Red Hydrogen Peroxide/Peroxidase Assay kit (Invitrogen molecular probe, A22188; Thermo Fisher Scientific Co. Ltd, Shanghai, China) following the manufacturer's directions. Absorbance was measured in a 96‐well microplate reader (Thermo Scientific Microplate Reader, Thermo Fisher Scientific Co. Ltd., Shanghai, China) at 560 nm, and the amount of H_2_O_2_ was calculated according to a standard curve. O^•^_2_^−^ histochemical staining was performed using NBT (Nitrotetrazolium Blue chloride) as in (Wu *et al*., 2017). Briefly, the leaf samples were spray‐inoculated with Guy11 (1 × 10^5^ mL^−1^ spores) or water. After 32 h the leaf samples were infiltrated with 50 mM sodium phosphate (pH 7.0) containing 0.05% NBT (Sigma) and 10 mM NaN_3_, and incubated at 37°C in the dark for 16 h. Leaf samples were then washed with bleaching solution (ethanol : acetic acid, 3 : 1) at 70°C for 30 min to elute the chlorophyll. The leaf sections were observed with a microscope (Zeiss Axio Imager A2), and quantification of O^•^_2_^−^ concentrations was measured by imagej software.

### qRT‐PCR

Four‐ to six‐leaf‐stage rice seedlings were spray‐inoculated or drop‐inoculated with Guy11 and mock at a concentration of 1 × 10^5^ spores ml^−1^, and samples were collected at 48 hpi. Total RNA was extracted using TRIzol reagent (Invitrogen) and was reverse transcribed to cDNA using the PrimeScript™ RT reagent Kit with gDNA Eraser following the manufacturer's instruction (TaKaRa Biotechnology, Dalian, China). qRT‐PCR was performed using SYBR Green mix (QuantiNova SYBR Green PCR Kit, Qiagen) and the indicated primers (Table S1). The rice *ubiquitin* (*UBQ*) gene was selected as an internal reference to normalize data.

### Enzymatic activity assay

In order to determine the activity of CSD protein in gel, the CSD protein extracts was assayed as reported previously (Davis, 1964; Shah & Nahakpam, 2012). Nondenaturing polyacrylamide gel electrophoresis (PAGE, 7.5% running gel and 3.5% stacking gel (acrylamide : bis‐acrylamide = 29 : 1)) was performed at 4°C using 0.01 M Tris‐glycine (pH 8.3) as electrode buffer. Twenty microliters of protein mixed with glycerol were loaded and electrophoretically run using a current of 25 mA per slab. After electrophoresis, the gels were rinsed with distilled water and incubated for 30 min in 2.5 mM NBT, then immersed in 1.17 × 10^−6^ M riboflavin for 20 min and removed to a petri dish for irradiance with a fluorescent lamp. Light exposure led to the development of the purple color of insoluble formazan throughout the gel, except for the locations where CSD was localized. For protein visualization, 20 μL of protein mixed with loading buffer were boiled and electrophoresed on 10% SDS‐PAGE gel, and the protein were dyed with Coomassie Brilliant Blue. The enzyme activities of CSD, SOD and CAT were detected using the Activity Assay Kits (A001‐4 for CSD and SOD, Njjcbio, Nanjing, China; BC0205 for CAT, Solarbio, Beijing, China). In brief, six‐leaf‐stage seedlings were spray‐inoculated with Guy11/mock at a concentration of 1 × 10^5^ spore mL^−1^, and 100 mg rice leaves were collected and powdered in liquid nitrogen and then homogenized with the extracting buffer. The extracts were centrifuged at 15 000 ***g*** for 10 min at 4°C and the supernatant were mixed with the provided reagents following the protocols in these kits, respectively, and detected by testing the absorbance of the final reaction mix with a 96‐well microplate reader (Thermo Scientific Microplate Reader) at specific wavelengths (SOD and CSD at 550 nm; CAT at 240 nm). The enzyme activity was calculated following the recommended formulae. For SOD, 1 unit of enzyme activity is defined as the absorbance change ratio when the absorbance is inhibited by 50% per gram fresh sample at 550 nm in 1 mL reaction buffer. In brief, SOD or CSD_(U/g FW)_  = [(*A*_(control)_ − *A*_(sample)_) / *A*_(control)_]/50% × [*V*_(total reaction volume)_ / *V*_(tested sample volume)_] × 2_(sample fold dilution) _/0.1 g mL^−1^_(sample concentration)_. For CAT, 1 unit of enzyme activity is defined as the activity of degrading 1 nM H_2_O_2_ per gram fresh sample per minute at 240 nm. In brief, CAT_(U/g FW)_ = [Δ*A* × 1×10^−4^ L_(total volume of the reaction system)_ /$4.36\times 10^{4}\;M\;{\mathrm{cm}}^{-1}{}_{\left(H_{2}O_{2}\;\mathrm{extinction}\;\mathrm{ratio}\right)}$ /0.5 cm_(96‐well plate light‐diameter)_ × 10^9^]/[(10 × 10^−6^ L_(the volume of the tested sample)_ /1 × 10^−3^ L_(the volume of total sample)_ × 0.1 g_(FW)_]/1 min_(reaction time)_ = 9180 × Δ*A*.

## Results

### miR398b downregulates SODX expression

**Figure 1:**
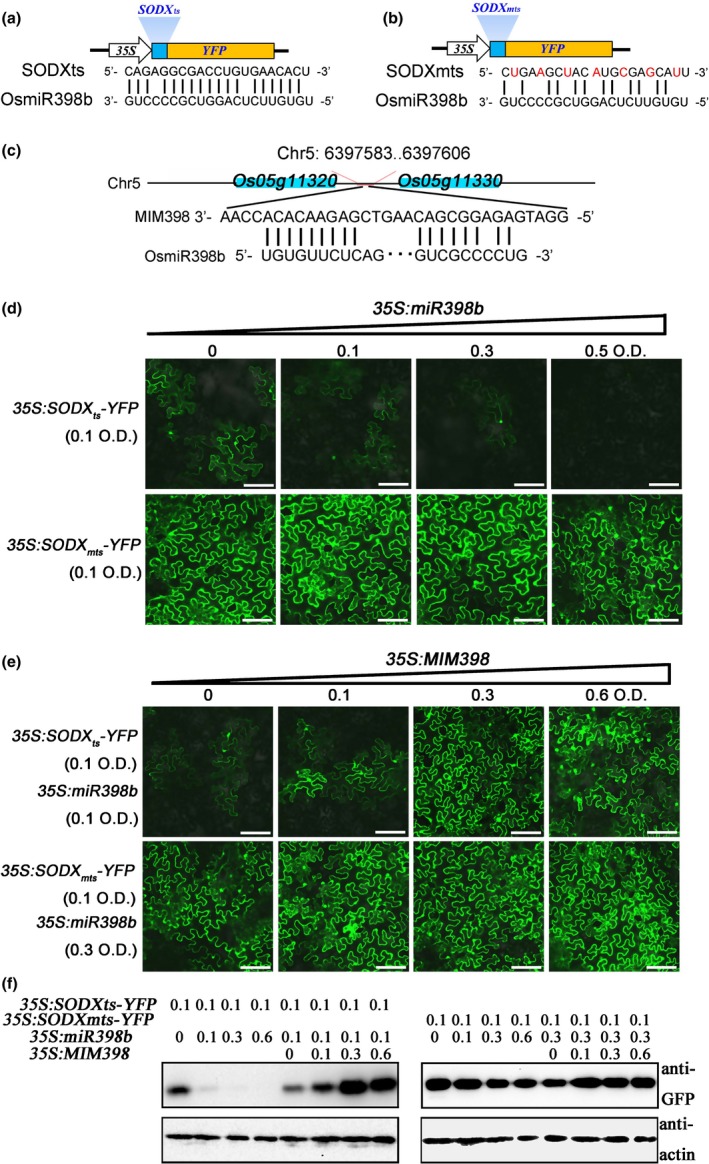
MiR398b represses the expression of *superoxidase dismutase* (*SOD*)*X*. (a) The construct of fused‐yellow fluorescent protein (YFP) with the target site of *SODX* (*SODXts‐YFP*) and alignment of miR398b with *SODX*_*ts*_ target site sequences. (b) The construct of fused‐YFP with the mutated target site of *SODX* (*SODXmts‐YFP*) and alignment of miR398b with *SODX*_*mts*_ target site sequences. The red letters indicate the mutated bases. (c) Alignment of miR398b with target mimicry of miR398 (MIM398) target site sequences. (d, e) Confocal images show the protein concentrations of SODXts‐YFP and SODXmts‐YFP. The indicated *SODXts‐YFP* and *SODXmts‐YFP* reporter constructs were transiently expressed alone or coexpressed with miR398b (d) or miR398b plus MIM398 (e) in *Nicotiana benthamiana* leaves using Agrobacterium‐mediated infiltration at the indicated optical density(OD)concentration. Bars, 50 μm. (f) Western blotting analysis shows the protein concentrations of SODXts‐YFP and SODXmts‐YFP. Protein extracts from the same amount of infiltrated leaves were subjected to Western blot analysis using anti‐green fluorescent protein (GFP) sera and anti‐actin sera, respectively. This experiment was repeated two times with similar results.

In order to confirm that *SODX* was repressed by miR398b, we made constructs expressing YFP fused with the putative target site of *SODX* at its 5′‐terminus (*35S:SODX*_*ts*_ *‐YFP*) (Fig. 1a) or with mutated target site (*35S:SODX*_*mts*_ *‐YFP*) that abolished recognition by miR398b (Fig. 1b). A construct expressing a target mimicry of miR398 (*MIM398*) also was created to act as a sponge and capture miR398, preventing silencing of its target (Wu *et al*., 2013; Fig. 1c). The YFP intensity and protein concentration expressed from *35S:SODX*_*ts*_ *‐YFP* was obviously lower when coexpressed with miR398b than when expressed alone in *N. benthamiana*, but recovered when coexpressed with *MIM398* (Fig. 1d,f). By contrast, the YFP concentration expressed from *35S:SODX*_*mts*_ *‐YFP* was unchanged when coexpressed with miR398b or miR398b plus MIM398 (Fig. 1e,f). These results indicate that miR398b represses *SODX* expression. Thus, miR398b suppresses its four target genes that belong to the SOD gene family.

### Mutants of miR398b target genes display different sensitivity to *M. oryzae*

In a previous report, we demonstrated that rice *OX398b* lines displayed higher resistance against *M. oryzae*. Because a miRNA functions through its target genes, we focused on functional characterization of the miR398b target genes and assessed the role of each target gene in blast disease‐resistance. We modified each of the four target genes using the CRISPR/Cas9 technology and identified two homozygous mutants for *CSD1* (*csd1‐1* AND *csd1‐3*), two for *CSD2* (*csd2‐1* and *csd2‐33*), two for *SODX* (*sodx‐5* and *sodx‐14*) and one for *CCSD* (*ccsd‐1*) (Fig. S1). Although *csd1‐1* carried a 3‐bp (CGG) deletion causing a threonine/serine substitution and a glycine deletion (Fig. S1a,b), the other six mutants carried insertions or deletions causing protein truncation (Fig. S1c–n). *csd1‐3* carried a 5‐bp deletion resulting in an early stop codon after amino acid residue (aa) 14 (glutamine; Fig. S1c,d); *csd2‐1* carried a G/A substitution and a 28‐bp deletion resulting in changes from aa 30 and led to an early stop after aa 76 (Isoleucine, Fig. S1e,f); *csd2‐33* carried a 2‐bp deletion resulting in changes starting at aa 34 and led to an early stop after aa 49 (arginine; Fig. S1g,h); *ccsd‐1* carried a 1‐bp insertion resulting in an early stop after aa 82 (Valine; Fig. S1i,j); *sodx‐5* carried a 5‐bp deletion resulting in changes starting at aa 35 and led to an early stop codon after aa 47 (arginine; Fig. S1k,l); *sodx‐14* carried a 1‐bp deletion resulting in changes starting at aa 72 and led to an early stop after aa 75 (histidine; Fig. S1m,n).

**Figure 2:**
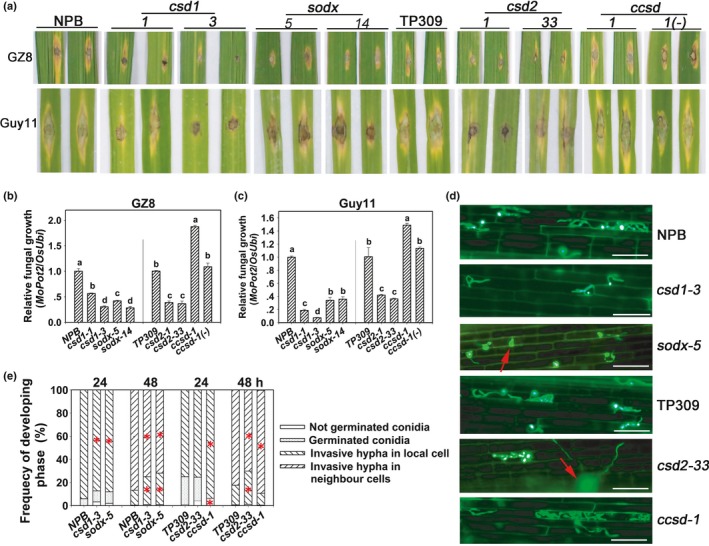
Mutations in miR398b target genes alters sensitivity to *Magnaporthe oryzae*. (a) Disease phenotypes of the indicated mutant lines (*Cu/Zn‐Superoxidase dismutase* (*csd*)1, *csd*2, *copper chaperone for superoxide dismutase* (*ccsd*), *superoxidase dismutase* (*sod*)*x*,* ccsd‐1* segregated azygous line (*ccsd‐1*(‐))) and their corresponding controls (Nipponbare (NPB), Taipei 309 (TP309)) at 5 d post‐inoculation (dpi) by *M. oryzae* strains GZ8 and Guy11. (b, c) Relative fungal biomass on the inoculated leaves of the inoculated lines at 5 dpi. The relative fungal biomass was measured by using the DNA amount of *M. oryzae Pot2* against the rice genomic ubiquitin DNA amount. Values are means ± SD of three replications. Different letters above the bars indicate significant differences at *P* < 0.01 as determined by a one‐way ANOVA followed by post hoc Tukey honest significant difference (HSD) analysis. (d) Representative epifluorescent microscopic images show the growth of the eGFP‐tagged *M. oryzae* strain GZ8 on sheath cells of the indicated mutant lines and the control lines at 36 h post‐inoculation (hpi), respectively. Red arrows indicate germinated but not invaded conidia. Bars, 50 μm. (e) Quantitative analysis of *M. oryzae* growth. More than 200 conidia in each line were analyzed. Significant differences between the corresponding wild‐type controls and mutant lines as determined by a one‐way ANOVA followed by post hoc Tukey HSD analysis are indicated: *, *P* < 0.05. All of the experiments were repeated two times with similar results.

**Figure 3:**
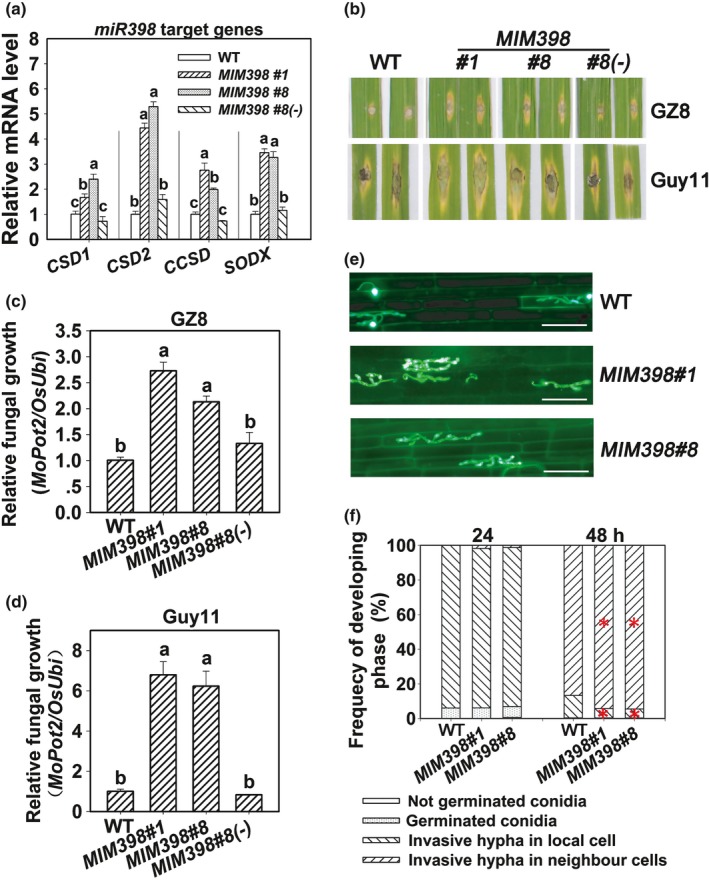
Overexpressing target mimicry of miR398b enhances sensitivity to *Magnaporthe oryzae*. (a) mRNA amounts of target genes in wild‐type (WT), target mimicry of miR398 (*MIM398*) and *MIM398#8* segregated azygous line (*MIM398#8*(‐)). The mRNA amounts were normalized to that in WT plants. Values are means of three replications. Error bars indicate SD. Different letters above the bars indicate significant differences at *P* < 0.01 as determined by a one‐way ANOVA followed by post hoc Tukey honest significant difference (HSD) analysis. (b) Disease phenotypes on leaves of the indicated lines upon *M. oryzae* strains GZ8 and Guy11 infection at 5 d post‐inoculation (dpi). (c, d) Relative fungal mass of GZ8 and Guy11 on the inoculated leaves of the indicated lines. The relative fungal mass was measured by using the DNA amount of *M. oryzae Pot2* against the rice genomic ubiquitin DNA amount. Values are means of three replications. Error bars indicate SD. Different letters above the bars indicate significant differences at *P* < 0.01 as determined by a one‐way ANOVA followed by post hoc Tukey HSD analysis. (e) Representative epifluorescent microscopic images show the growth of GZ8 at 36 h post‐inoculation (hpi) on sheath cells of the indicated transgenic lines and the WT plants, respectively. Bars, 50 μm. (f) Quantitative analysis of *M. oryzae* growth at the indicated time points. More than 200 conidia in each line were analyzed. Significant differences at between the WT and MIM398 lines as determined by a one‐way ANOVA followed by post hoc Tukey HSD analysis are indicated: *, *P* < 0.05. All of the experiments were repeated twice with similar results.

We then assessed the sensitivity of these mutants to the rice blast fungus via punch‐ and spray‐inoculation with different virulent strains. Punch‐inoculation results showed that *csd1*,* csd2* and *sodx* are less susceptible to *M. oryzae* strains GZ8 and Guy11, displaying smaller disease lesions and significantly lower fungal growth than that of their corresponding WT control (Fig. 2a–c), However, *ccsd‐1* displayed enhanced susceptibility to both strains, whereas the *ccsd‐1* segregated azygous control line, *ccsd‐1*(‐), showed similar fungal growth as the WT (Fig. 2a–c). Similarly, spray‐inoculation with another *M. oryzae* strain 089 showed that *OX398b*,* csd1*,* csd2* and *sodx* were less susceptible, but *ccsd* was more susceptible than WT control (Fig. S2a,b). To understand how *csd1*,* csd2* and *sodx* were less susceptible and *ccsd* was more susceptible, we observed the fungal infection progress by quantifying the formation and expansion of invasive hyphae in leaf sheath cells. Consistent with the disease phenotypes, the infection progress is significantly delayed in *csd1*,* csd2* and *sodx*, but enhanced in *ccsd* compared with the corresponding WT control (Fig. 3d,e; Table S3). In Nipponbare (NPB), >93.9% of inoculated spores formed invasive hyphae at 24 hpi and > 86.6% of them expanded into neighbor cells at 48 hpi; whereas, in both *csd1‐3* and *sodx‐5* lines, < 88.1% spores formed invasive hyphae at 24 hpi and < 75.1% of them expanded into neighbor cells at 48 hpi (Fig. 2d,e; Table S3). In TP309, > 74.9% spores formed invasive hyphae at 24 hpi and > 82.4% of them expanded into neighbor cells at 48 hpi; however, in *csd2‐33* < 70.2% expanded into neighbor cells at 48 hpi, although no difference was observed at 24 hpi (Fig. 2d,e; Table S3). Conversely, in *ccsd‐1*, 93.6% spores formed invasive hyphae at 24 hpi and > 88.9% of them extended into neighbor cells at 48 hpi (Fig. 2d,e; Table S3). These observations indicate that different target genes of miR398b play different roles in blast disease‐resistance. Although *CSD1*,* CSD2* and *SODX* may negatively regulate resistance, *CCSD* may be required for rice blast disease‐resistance in rice.

### Overexpression of miR398b target genes enhances rice sensitivity to *M. oryzae*

In order to achieve upregulation of all the target genes simultaneously, we generated transgenic lines expressing *MIM398*. All of the *MIM398* lines express significantly higher mRNA amounts of the four target genes of miR398b, whereas, the segregated azygous control (*MIM398#8(‐)*) retains WT mRNA amounts (Fig. 3a). Moreover, *MIM398* lines are more susceptible to GZ8 and Guy11, with larger disease lesions and significantly more fungal growth than WT control (Fig. 3b–d); whereas *MIM398#8(‐)* displays the similar susceptibility to both strains (Fig. 3b–d). Consistently with the macroscopic disease phenotypes, the infection progress is significantly enhanced in *MIM398* lines compared with WT control (Fig. 3e,f; Table S3). In NPB, > 93.9% spores formed invasive hyphae at 24 hpi and < 86.7% of spore‐formed invasive hypha expanded into neighbor cells at 48 hpi; however, in *MIM398* lines, > 94.2% of them expanded into neighbor cells at 48 hpi, although no difference was observed at 24 hpi (Fig. 3e,f; Table S3). These results indicate that upregulation of the miR398b target genes facilitates the growth of *M. oryzae* presumably via suppressing rice immunity, but miR398b positively contributes to blast resistance by silencing them.

**Figure 4:**
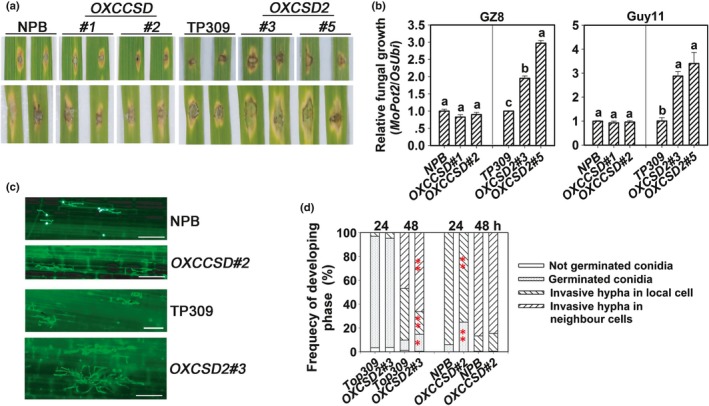
Overexpressing Cu/Zn‐Superoxidase Dismutase (CSD)2 enhances sensitivity to *Magnaporthe oryzae*. (a) Disease phenotypes on leaves of the inoculated lines 5 d post‐inoculation (dpi) by *M. oryzae* strains GZ8 and Guy11 infection, separately. (b) The relative fungal mass on the inoculated leaves of the indicated lines at 5 dpi. The relative fungal mass was measured by using the DNA amount of *M. oryzae Pot2* against the rice genomic ubiquitin DNA amount. Values are means ± SD of three replications. Different letters above the bars indicate significant differences at *P* < 0.01 as determined by a one‐way ANOVA followed by post hoc Tukey honest significant difference (HSD) analysis. (c) Representative epifluorescent microscopic images show the growth of GZ8 at 36 h post‐inoculation (hpi) on sheath cells of the indicated transgenic lines (OXCCSD,* Copper Chaperone for Superoxide Dismutase* (*CCSD*) overexpressing line; OXCSD2, *CSD2* overexpressing line) and the corresponding wild‐type (WT) controls (NPB, Nipponbare. TP309, Taipei 309), respectively. Bars, 50 μm. (d) The quantitative analysis of *M. oryzae* growth at indicated time points. More than 200 conidia in each line were analyzed. Significant differences between the corresponding WT controls and overexpression lines as determined by a one‐way ANOVA followed by post hoc Tukey HSD analysis: *, *P* < 0.05; **, *P* < 0.01. All of the experiments were repeated two times with similar results.

In order to further confirm the roles of target genes in blast disease‐resistance, we generated transgenic lines overexpressing miR398b‐insensitive *CCSD*_*m*_ *‐GFP* (*OXCCSD*) and *CSD2*_*m*_ *‐GFP* (*OXCSD2*). *CCSDm‐GFP* contains six mismatches in the miR398b target site, and *CSD2*_*m*_ *‐GFP* excludes the miR398b target site located in the 5′‐UTR of CSD2 (Fig. S3a,b). Higher *CSD2* and *CCSD* mRNA amounts were detected (Fig. S3c,e) and the fusion proteins are present in the nucleus and the cytoplasm (Fig. S3d,f). By contrast to the *csd2* mutants that are less susceptible, the *OXCSD2* lines are more susceptible to *M. oryzae*, boasting larger disease lesions and more fungal growth (Fig. 4a,b), and displaying a significantly higher ratio of invasive hyphae from the primary infected cells expanding into neighbor cells at 48 hpi (Fig. 4c,d; Table S3). These observations indicate that CSD2_m_‐GFP is functional and negatively regulates blast disease‐resistance. The *OXCCSD* lines display similar susceptibility and disease lesions to WT (Fig. 4a,b), although they show a significantly lower ratio of invasive hyphae at local cells at 24 hpi (Fig. 4c,d; Table S3). Further investigation is required to clarify whether CCSD_m_‐GFP is functional or over‐accumulation of CCSD does not enhance rice blast resistance.

### miR398b and its target genes regulate ROS concentration upon *M. oryzae* infection

**Figure 5:**
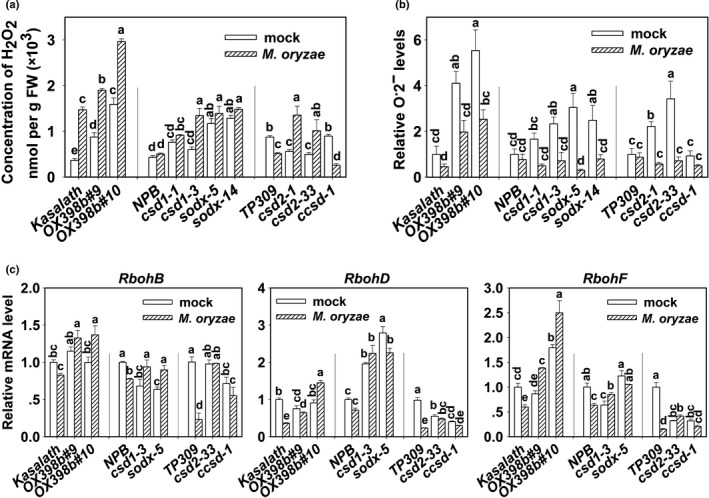
Overexpressing miR398b or mutations in target genes affected reactive oxygen species (ROS) concentration upon *Magnaporthe oryzae* infection. (a, b) Quantification of hydrogen peroxide (H_2_O_2_) (a) and O^•^_2_^−^ (b) concentrations in leaves of the wild‐type (WT) (Kasalath, Nipponbare (NPB), Taipei 309 (TP309)) and indicated lines (miR398b overexpressing lines (*OX398b*), mutant of *Cu/Zn‐Superoxidase Dismutase* (*csd*)1, *csd*2, mutant of *Copper Chaperone for Superoxide Dismutase* (*ccsd*), mutant of *Superoxidase Dismutase* (*sod*)x) with Guy11/mock treatment at 48 h post‐inoculation (hpi), respectively. Values are means of three replications. Error bars indicate SD. Different letters above the bars indicate significant differences at *P* < 0.01 as determined by a one‐way ANOVA followed by post hoc Tukey honest significant difference (HSD) analysis. (c) Quantitative reverse transcription polymerase chain reaction (qRT‐PCR) data showing the expression pattern of the indicated NADPH oxidase genes (*Respiratory burst oxidase homologs* (*Rboh*) B, *Rboh*D and *Rboh*F) in the indicated lines upon Guy11/mock treatment at 48 hpi. Relative mRNA amounts were normalized to that in WT mock samples. Values are means of three replications. Error bars indicate SD. Different letters above the bars indicate significant differences at *P* < 0.01 as determined by a one‐way ANOVA followed by post hoc Tukey HSD analysis. All of the experiments were repeated two times with similar results.

Accumulation of H_2_O_2_ is a common defense response to *M. oryzae* in rice (Chen L. *et al.*, 2012; Chen M. *et al*., 2012; Shimono *et al.*, 2012; Wang *et al.*, 2016). To test whether H_2_O_2_ accumulation contributes to the observed resistance phenotypes, we examined H_2_O_2_ concentrations in all tested lines. Consistently with the disease phenotypes, upon Guy11 infection, H_2_O_2_ concentrations increased significantly higher in *OX398b*,* csd1*,* csd2* and *sodx* compared to those in their corresponding WT controls, but decreased and significantly lower in *ccsd* (Figs 5a, S4). Although *MIM398* displayed higher H_2_O_2_ concentrations with mock treatment (Fig. S5a,b), upon Guy11 infection, *MIM398*,* OXCSD2* and *OXCCSD* all showed lower or similar H_2_O_2_ concentrations compared to their corresponding WT controls (Fig. S5a,b). These results indicate that miR398b positively regulates H_2_O_2_ accumulation by repressing CSD1, CSD2 and SODX in rice infected with *M. oryzae*, and overexpressing target genes does not positively contribute to blast‐induced H_2_O_2_ accumulation.

Because O^•^_2_^−^ is the precursor of H_2_O_2_, we next examined its concentration by Nitroblue tetrazolium (NBT) staining (Wu *et al*., 2017). Upon mock treatment, O^•^_2_^−^ concentrations in *OX398b*,* csd1*,* csd2* and *sodx*, but not in *ccsd*, were generally higher compared to those in their corresponding WT controls; in particular, the O^•^_2_^−^ concentration in *OX398b* is markedly higher (Figs 5b, S4). Conversely, *MIM398*,* CCSD* and *CSD2* showed lower or similar O^•^_2_^−^ concentrations (Fig. S5a,c). These data indicate that silencing of *CSD1*,* CSD2* and *SODX* by miR398b positively contributes to O^•^_2_^−^ concentrations, whereas mutation of *CCSD* or overexpressing target genes do not lead to significant alteration of O^•^_2_^−^ concentrations. More interestingly, the O^•^_2_^−^ concentrations markedly decreased upon Guy11 infection in *OX398b*,* csd1*,* csd2* and *sodx* lines, but only slightly changed in *ccsd* and target gene overexpressing lines (Figs 5b, S5a,c), suggesting that overexpression of miR398b, or mutations in *CSD1*,* CSD2* and *SODX* positively regulated the blast infection‐promoted conversion of O^•^_2_^−^ to H_2_O_2_, whereas overexpression of target genes had little effect on the conversion process.

NADPH oxidase is the key enzyme catalyzing the production of O^•^_2_^−^ (Kaur *et al*., 2014). We then monitored the mRNA amounts of three NADPH oxidase genes, *RbohB* (Nagano *et al*., 2016), *RbohD* and *RbohF* (Jang *et al*., 2012). Guy11 infection clearly decreased their RNA amounts in all three WT rice varieties – Kasalath, NPB and TP309. By contrast, upon Guy11 infection, the mRNA amounts for those genes in *OX398b*,* csd1*,* csd2* and *sodx* were all higher than in WT control plants (Fig. 5c–e). These results indicate that upon *M. oryzae* infection, overexpression of miR*398b* or mutations in *CSD1*,* CSD2* and *SODX* leads to higher ROS concentrations, resulting enhanced blast disease‐resistance.

### miR398b and its target genes regulate CSD expression and activity

**Figure 6:**
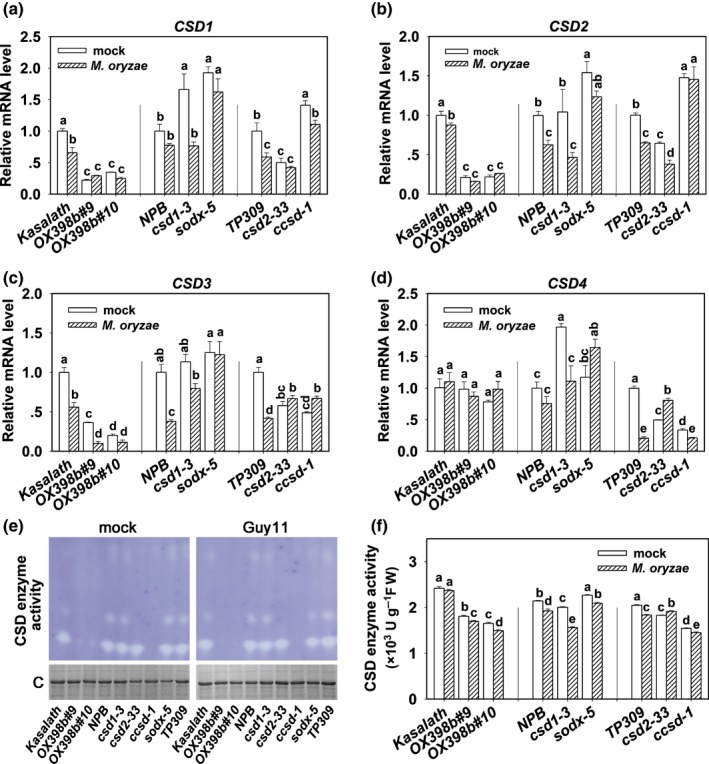
Overexpressing miR398b or mutations in target genes affected Cu/Zn‐Superoxidase Dismutase (CSD) concentrations and enzyme activity. (a–d) Quantitative reverse transcription polymerase chain reaction (qRT‐PCR) data showing the expression pattern of the *CSD* subfamily genes in wild‐type (WT) (Kasalath, Nipponbare (NPB), Taipei 309 (TP309)) and indicated transgenic lines with *Magnaporthe oryzae* strains Guy11 or mock infection at 48 h post‐inoculation (hpi). Relative mRNA amount was normalized to that in WT mock samples. Values are means of three replications. Error bars indicate SD. Different letters above the bars indicate significant differences at *P* < 0.01 as determined by a one‐way ANOVA followed by post hoc Tukey honest significant difference (HSD) analysis. (e) The CSD enzyme activity in gel with Guy11/mock treatment. Coomassie (C) dying indicates the protein loading. (f) The enzyme activity of CSD in indicated lines with Guy11/mock treatment. Values are means of four replications. Error bars indicate SD. Different letters above the bars indicate significant differences at *P* < 0.01 as determined by a one‐way ANOVA followed by post hoc Tukey HSD analysis. All of the experiments were repeated two times with similar results.

Reduction of O^•^_2_^−^ into H_2_O_2_ is primarily conducted by SOD isoenzymes (Fridovich, 1995; Gill & Tuteja, 2010). In rice, the SOD gene family contains fifteen members, including four CSDs, four SODs and another seven related chaperones including CCSD (Nath *et al*., 2014). Among the 15 members, the four CSDs were identified as *CSD1*,* CSD2*,* CSD3* and *CSD4* (Fig. S6). We first tested whether the expression levels of these CSDs were altered in *OX398b* and the mutants. As shown in Fig. 6a–d, *OX398b* lines display significantly decreased levels of *CSD1*,* CSD2* and *CSD3* with or without blast treatment, indicating that overexpression of miR398b leads to lower CSD expression; upon Guy11 infection, nearly all mutants lines displayed less or unchanged mRNA amounts of the four *CSDs*, suggesting that *M.oryzae* suppressed CSD expression.

We next examined CSD enzyme activity in these lines by photochemical method (Shah & Nahakpam, 2012) and spectrophotometry (Wu *et al*., 2017). Without blast treatment, *OX398b* displayed a significant reduction of bands in the gels, indicative of less CSD enzyme activity; *ccsd* lost major bands (Fig. 6e), suggesting that CCSD was required for CSD activity. Consistently, *OX398b*,* csd1*,* csd2* and *ccsd* showed reduced CSD enzyme activity compared to their corresponding WT controls, whereas *sodx* showed slightly higher CSD activity (Fig. 6f). These results indicate that CSD1, CSD2 and CCSD positively, whereas SODX negatively, regulate CSD enzyme activity. Upon Guy11 infection, all tested lines displayed significantly lower CSD activity than the mock inoculation, except *csd2* (Fig. 6f), indicating that *M. oryzae* suppresses CSD enzyme activity. However, the blast‐suppressed CSD activity contradicts the blast‐induced higher H_2_O_2_ concentrations in *OX398b* and mutant lines except *ccsd* (Fig. 5), indicating that some other SOD family members are possibly involved in miR398b‐regulated H_2_O_2_ accumulation upon *M. oryzae* infection.

We also tested the mRNA amounts of the four CSD genes and CSD enzyme activity in the target gene overexpressing lines. Upon mock treatment, whereas *MIM398* displayed higher CSD enzyme activity associated with higher mRNA amount of CSD1 and CSD2 compared to the WT control, *OXCSD2* showed higher activity accompanied by a higher CSD2 mRNA amount (Fig. S7a–e). Upon Guy11 infection, although all tested lines displayed decreased CSD activity, *MIM398* and *OXCCSD* also showed higher activity compared to the WT control (Fig. S7e). These results confirm that rice blast suppresses CSD expression and enzyme activity, and imply that overexpression of miR398b target genes or overexpression of CSD2 alone enhances CSD enzyme activity.

### miR398b and its target genes regulate SOD concentration and enzyme activity

**Figure 7:**
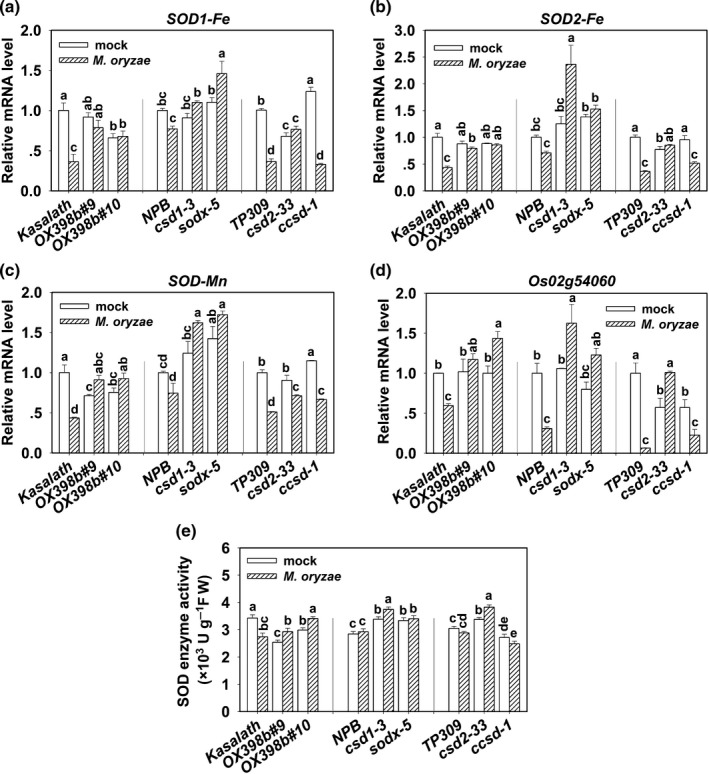
Overexpressing miR398b or mutations in target genes affected total Superoxidase Dismutase (SOD) concentrations and enzyme activity. (a–d) Quantitative reverse transcription polymerase chain reaction (qRT‐PCR) data showing the expression pattern of the SOD family genes in wild‐type (WT) (Kasalath, Nipponbare (NPB), Taipei 309 (TP309)) and indicated transgenic lines upon *Magnaporthe oryzae* strains Guy11 or mock treatment at 48 h post‐inoculation (hpi). Relative mRNA amount was normalized to that in WT mock samples at 48 hpi. Values are means of three replications. Error bars indicate SD. Different letters above the bars indicate significant differences at *P* < 0.01 as determined by a one‐way ANOVA followed by post hoc Tukey honest significant difference (HSD) analysis. (e) The total SOD enzyme activity in transgenic lines and WT plants with Guy11 or mock treatment at 48 hpi. Results are the means of four replicates. Error bars indicate SD. Different letters above the bars indicate significant differences at *P* < 0.01 as determined by a one‐way ANOVA followed by post hoc Tukey HSD analysis. All of the experiments were repeated two times with similar results.

In order to assess whether other SOD family genes contribute to the elevated H_2_O_2_ concentrations in *OX398b*,* csd1*,* csd2* and *sodx*, we examined the mRNA amount of the other SOD family members and total SOD enzyme activity. Our data showed that upon *M. oryzae* infection, the mRNA amounts of *SOD1‐Fe*,* SOD2‐Fe*,* SOD‐Mn*, and a SOD family chaperone, *Os02g54060*, were significantly decreased in WT and *ccsd*, but unchanged or even increased in *OX398b*,* csd1*,* csd2* and *sodx*, although *SOD‐Mn* was decreased in *csd2* (Fig. 7a–d). Consistently, total SOD enzyme activity was upregulated in *OX398b*,* csd1*,* csd2* and *sodx*, but was downregulated or remained unchanged in WT control plants and the *ccsd* mutant upon Guy11 infection (Fig. 7e). By contrast, the *MIM398*,* OXCSD2* and *OXCCSD* lines showed lower or unchanged mRNA amount of these genes and unchanged or decreased SOD enzyme activity upon Guy11 infection compared to their corresponding WT controls (Fig. S8a–e). These data indicate that upon *M. oryzae* infection, miR398b silences target genes *CSD1, CSD2* and *SODX*, which in turn triggers upregulation of other *SOD* family members resulting in higher total SOD enzyme activity, leading to higher H_2_O_2_ production and enhanced blast disease‐resistance.

Because the observed H_2_O_2_ accumulation could be due to reduced H_2_O_2_ degradation in *OX398b* and the target gene mutants, we tested the activity of the main H_2_O_2_ degradation enzyme, catalase (CAT) (Kim *et al*., 2017). We found no obvious differences between the transgenic lines and WT upon *M. oryzae* infection (Fig. S9), indicating that CAT was not involved in miR398b‐mediated resistance to *M. oryzae* by catalyzing the conversion of H_2_O_2_ into H_2_O and O_2_.

## Discussion

**Figure 8:**
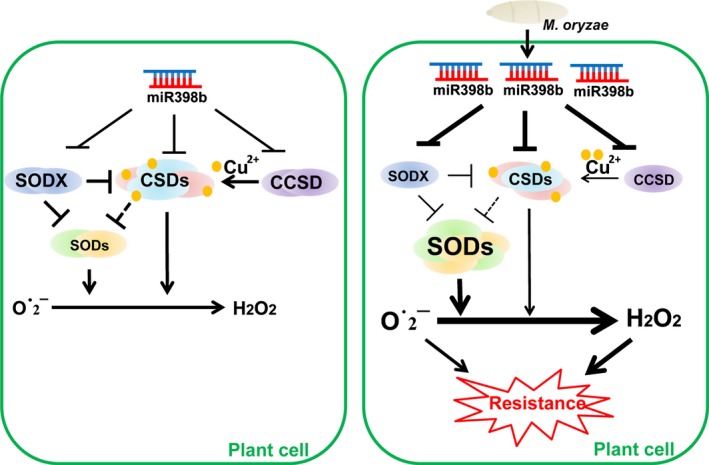
A model of miRNA398b functions in plant immunity to *Magnaporthe oryzae*. Without pathogen infection, miR398b‐regulated Cu/Zn‐Superoxidase Dismutase (CSD)1, CSD2, Superoxidase Dismutase (SOD)X and Copper Chaperone for Superoxide Dismutase (CCSD) maintain a basal CSD and total SOD enzyme activity level. CCSD positively contributes to CSD activity by transferring coppers to CSD protein. By contrast, SODX negatively regulates CSD and SOD activity by unknown mechanisms. A compensatory regulation mechanism exists between CSD and other SODs to maintain the hydrogen peroxide (H_2_O_2_) concentrations for normal metabolism in cells. Upon *M. oryzae* infection, the elevated miR398b accumulation represses the expression of CSDs/SODX/CCSD. On the one hand, the decreased CSDs and CCSD leads to lower CSD enzyme activity, which in turn results in higher expression and activity of other SODs by the unknown compensatory regulation mechanism. On the other, the decreased SODX compromises the repression on CSDs and SODs, and results in higher total SOD activity. As a result, the enhanced total SOD enzyme activity generates more H_2_O_2_ synthesis and results in enhanced resistance. Arrows indicate positive regulation and blunt‐ended bars indicate inhibition. The dotted lines indicate unidentified regulation between CSDs and SODs.

In a previous paper, miR398b is reported to positively regulate rice basal defense against *M. oryzae*, which is different from its negative role in defense in Arabidopsis (Li *et al*., 2010a, 2014). Here, we further demonstrated that miR398b coordinates multiple pathways to boost hydrogen peroxide (H_2_O_2_) production by regulating the expression of Cu/Zn Superoxidase Dismutase (CSD)1, CSD2, Copper Chaperone for Superoxide Dismutase (CCSD) and Superoxidase DismutaseX (SODX) upon *M. oryzae* infection. Functional analysis of these mutants indicates that *CSD1, CSD2, CCSD* and *SODX* play different roles in regulation of H_2_O_2_ concentrations and host resistance (Figs 2, 3, 4, 5). Mutations in *SODX* lead to higher CSD and total SOD activity, whereas mutations in *CCSD* compromise CSD activity (Figs 6, 7). Furthermore, mutations in *CSD1* and *CSD2* compromise CSD activity but lead to increased expression of other SODs and total SOD activity (Figs 6, 7), indicating that some compensatory regulation mechanism exists between CSDs and other SOD family members. Based on these data, we propose a working model to explain how miR398b boosts H_2_O_2_ production via multiple SODs (Fig. 8). Although CCSD is required for CSD activity to transfer copper to CSDs, SODX negatively regulates the activity of CSD and other SODs, such as SOD‐Fe and SOD‐Mn, by unknown mechanisms. These might be certain compensatory regulation or balance between CSDs and SODs. Without *M. oryzae* infection, miR398b regulates the expression of CSD1, CSD2, SODX and CCSD to maintain a basal CSD and total SOD activity level for normal H_2_O_2_ concentrations and related metabolic activity in rice cells (Fig. 8). Upon *M. oryzae* infection, the increased accumulation of miR398b represses the expression of all target genes. On the one hand, reduced accumulation of CSDs and CCSD leads to decreased CSD activity, which in turn results in increased expression of other SODs and higher total SOD activity by certain compensatory regulation mechanisms to enhance conversion of O^•^_2_^−^ into H_2_O_2_; on the other, the reduction of SODX compromises the repression on CSD and SODs, resulting in higher total SOD activity (Fig. 8). In summary, upon *M. oryzae* infection, miR398b overexpression enhances resistance by repressing target genes to upregulate total SOD concentrations and enzyme activity to generate more H_2_O_2_ (Fig. 8). Therefore, our data provide new insight into the roles of miR398b in regulation of rice disease resistance.

It is possible that miR398b activates a coordinated regulation network of CSD and SOD to positively regulate host resistance to rice blast disease. Without pathogen infection, *OX398b* displayed significantly lower CSD (Fig. 6) and total SOD activity compared to WT control (Fig. 7). However, upon *M. oryzae* infection, *OX398b* showed higher expression of other SOD genes and total SOD activity (Fig. 7) associated with higher H_2_O_2_ accumulation (Fig. 5), suggesting that miR398b upregulates total SOD activity to fight against *M. oryzae* invasion via upregulation of H_2_O_2_ production. By contrast, mutations of *CSD1* and *CSD2*, respectively, resulted in lower CSD enzyme activity (Fig. 6e,f), whereas overexpression of *CSD2* led to higher CSD enzyme activity (Fig. S7e), suggesting CSD1 and CSD2 are important CSD enzymes and positively contribute to CSD activity. However, upon *M. oryzae* infection, both *csd1* and *csd2* showed increased SOD‐Fe and SOD‐Mn expression, and total enzyme activity (Fig. 7a–e), resulting in increased H_2_O_2_ concentration (Fig. 5) and enhanced host resistance (Figs 2, S2), suggesting the existence of compensatory regulation mechanisms that perceive ROS concentrations upon *M. oryzae* infection and result in increased expression and activity of other SOD family members. However, how the decreased expression and activity of CSD triggers the compensatory regulation mechanisms needs further investigation. Interestingly, similar compensatory regulation mechanism possibly exists in other plants. For example, miR398 is upregulated, whereas *CSD1* is downregulated in response to water deficit‐tolerance in *Medicago truncatula*, (Trindade *et al*., 2010), and miR398 positively contributes to heat tolerance by reducing transcripts of *CSD1*,* CSD2* and *CCS* in Arabidopsis (Guan *et al*., 2013; Lu *et al*., 2013).

The two target genes of miR398b, *CCSD* and *SODX*, act antagonistically in regulating CSD and total SOD activity. CCSD is a chaperone of CSD, and *ccsd* showed lower CSD (Fig. 6e,f) and total SOD enzyme activity (Fig. 7e), indicating that CCSD is important for the CSD activity. However, *OXCCSD* displayed unaffected fungal growth, CSD and total SOD activity compared to wild‐type plants (Figs 3, 8, S7). One possible explanation is that CCSD is a CSD chaperone protein, not a CSD or SOD protein, and overexpression of CCSD cannot enhance CSD protein concentrations, or activity. The other possible explanation is that the GFP fusion disturbs the normal function of CCSD in the transgenic lines. SODX, an uncharacterized protein, is located near SOD‐Fe and SOD‐Mn in the phylogenetic tree (Fig. S6). We confirmed that its expression was repressed by miR398b (Fig. 1). Mutation of this gene in two different sites resulted in increased host resistance and higher ROS concentrations (Figs 2, 5, S2), indicating that SODX negatively regulates rice resistance. *sodx* displayed higher CSD concentration and activity (Fig. 7), as well as higher expression of SOD‐Fe and SOD‐Mn, and higher total SOD activity (Fig. 7), implying that SODX suppresses both CSDs and other SODs. However, the biochemical function of SODX, and how SODX negatively regulates CSD and total SOD activity needs further investigation.

miR398b mediates a coordinated and balanced regulatory module via CCSD, CSD and SODX. Overexpression of *OX398b* led to a significant reduction of CCSD concentration, but enhanced resistance to the blast disease, which appears to contradict the susceptibility of the *ccsd* mutant. There are several possible reasons for this paradox. First of all, *OX398b* reduced, but did not completely abolish the expression of CCSD (Li *et al*., 2014), CSD1 and CSD2 (Fig. 6a,b), indicating that *OX398b* retained certain CCSD‐dependent CSD activity. Second, miR398b also repressed SODX, releasing the SODX‐mediated suppression of CSD and SOD, resulting in higher CSD and SOD enzyme activity. Third, upon *M. oryzae* infection, the lower accumulation of CSD1 and CSD2 triggered a compensatory regulation mechanism resulting in higher expression of the other SOD family members and higher SOD activity. Thus, miR398b integratively upregulates the total SOD activity upon *M. oryzae* infection.

The SODs catalyze the conversion of O^•^_2_^−^ into H_2_O_2_, which acts as a signal molecule to trigger resistance to various biotic and abiotic stresses (Quan *et al*., 2008; Kaur *et al*., 2016; Saxena *et al*., 2016). However, the excess H_2_O_2_ results in the occurrence of oxidative stress and leads to programmed cell death (PCD), which is harmful to plant development (Quan *et al*., 2008). Under this scenario, miR398b integratively regulates the activity of total SOD at certain levels to control moderate H_2_O_2_ accumulation. Upon *M. oryzae* infection, increased miR398b levels result in lower CSD1, CSD2 and CCSD concentrations and lower CSD activity, which is disadvantageous for rice to accumulate more H_2_O_2_ and fight against blast disease. In turn, the lower CSD activity triggers higher expression of other SOD family members, leading to higher total SOD activity. Such moderate compensatory regulation is based on the decreased CSD concentrations and avoids overaccumulation of H_2_O_2_, thereby boosting resistance in as well as avoiding unnecessary harm to rice.

In conclusion, upon *M. oryzae* infection, silencing of *CSD1* and *CSD2* by miR398b results in higher H_2_O_2_ concentrations associated with higher total SOD activity and greater resistance to infection; silencing of *SODX* leads to higher H_2_O_2_ concentrations accompanied by higher activity of both CSDs and SODs, whereas silencing of *CCSD* leads to lower H_2_O_2_ concentrations associated with lower activity of CSDs, which in turn triggers higher expression of other SODs. Therefore, the output of multiple SODs contributes to the miR398b‐regulated rice immunity against the blast fungus *M. oryzae*. Future research is needed to dissect the function of each member of the SOD family and their genetic interactions.

## Author contributions

YL, X‐LC and W‐MW designed the experiments; YL, X‐LC, YZ, K‐NZ, X‐MY, HW, Z‐YX, Y‐PZ, J‐QZ and W‐MW performed the experiments; YL, X‐LC, JW, O‐XD, X‐WC and W‐MW analyzed the data; YL, MC and W‐MW wrote the paper. J‐HZ, L‐LZ, G‐BL, JF, X‐QC and X‐JWu discussed the results and commented on the manuscript; and YL and X‐LC contributed equally to this work.

## Supporting information

Please note: Wiley Blackwell are not responsible for the content or functionality of any Supporting Information supplied by the authors. Any queries (other than missing material) should be directed to the *New Phytologist* Central Office.

**Fig. S1** Mutation sites of miR398b target genes in mutant lines.
**Fig. S2** Overexpressing miR398b or mutations in target genes alters sensitivity to *M. oryzae*.
**Fig. S3** The mRNA and protein concentrations of CSD2 and CCSD increases in overexpressing lines.
**Fig. S4** Representative leaf sections from the *OX398b* and mutant lines show the accumulation of H_2_O_2_ and O^•^_2_^−^.
**Fig. S5** Overexpressing MIM398 or overexpression of CSD2 does not upregulate ROS accumulation upon *M. oryzae* infection.
**Fig. S6** Phylogenetic tree of SOD family members in rice.
**Fig. S7** Overexpressing MIM398 or overexpression of miR398b target genes upregulate CSD accumulation and enzyme activity upon *M. oryzae* infection.
**Fig. S8** Overexpressing MIM398 or overexpression of miR398b target genes does not upregulate total SOD enzyme activity upon *M. oryzae* infection.
**Fig. S9** Overexpressing miR398b or mutations in target genes does not affect CAT enzyme activity.
**Methods S1** H_2_O_2_ measurement.
**Table S1** Primers used in this study.
**Table S2** Guide RNAs and target sites used in CRISPR/Cas9 technology.
**Table S3** Quantitative analysis of *M. oryzae* growth at the indicated time points.
