# Supplementary material for: Osa‐miR398b boosts H2O2 production and rice blast disease‐resistance via multiple superoxide dismutases
Source: New Phytol. 2019 Feb 6;222(3):1507–22. doi: 10.1111/nph.15678 (PMC6593823; doi:10.1111/nph.15678)
Supplement: Supplementary file 1 — Fig. S1 Mutation sites of miR398b target genes in mutant lines. Fig. S2 Overexpressing miR398b or mutations in target genes alters sensitivity to M. oryzae. Fig. S3 The mRNA and protein concentrations of CSD2 and CCSD increases in overexpressing lines. Fig. S4 Representative leaf sections from the OX398b and mutant lines show the accumulation of H2O2 and O• 2 −. Fig. S5 Overexpressing MIM398 or overexpression of CSD2 does not upregulate ROS accumulation upon M. oryzae infection. Fig. S6 Phylogenetic tree of SOD family members in rice. Fig. S7 Overexpressing MIM398 or overexpression of miR398b target genes upregulate CSD accumulation and enzyme activity upon M. oryzae infection. Fig. S8 Overexpressing MIM398 or overexpression of miR398b target genes does not upregulate total SOD enzyme activity upon M. oryzae infection. Fig. S9 Overexpressing miR398b or mutations in target genes does not affect CAT enzyme activity. Methods S1 H2O2 measurement. Table S1 Primers used in this study. Table S2 Guide RNAs and target sites used in CRISPR/Cas9 technology. Table S3 Quantitative analysis of M. oryzae growth at the indicated time points. [file NPH-222-1507-s001.pdf]

New Phytologist **Supporting Information**

Article title: **Osa-miR398b Boosts H2O2 Production and Rice Blast Disease-Resistance via Multiple Superoxide Dismutases**

Authors: Yan Li, Xiao-Long Cao, Yong Zhu, Xue-Mei Yang, Kai-Ni Zhang, Zhi-Yuan Xiao, He Wang, Jing-Hao Zhao, Ling-Li Zhang, Guo-Bang Li, Ya-Ping Zheng, Jing Fan, Jing Wang, Xiao-Qiong Chen, Xian-Jun Wu, Ji-Qun Zhao, Oliver Xiaoou Dong, Xue-Wei Chen, Mawsheng Chern, Wen-Ming Wang

The following Supporting Information is available for this article:

Methods S1 H_2O_2measurement

Fig. S1 Mutation sites of miR398b target genes in mutant lines.

Fig. S2 Overexpressing miR398b or mutations in target genes alters sensitivity to *M. oryzae*.

Fig. S3 The mRNA and protein levels of CSD2 and CCSD increases in overexpressing lines.

Fig. S4 Representative leaf sections from the *OX398b* and mutant lines show the accumulation of H_2O_2 and $O{\cdot}_2^-$.

Fig. S5 Overexpressing MIM398 or overexpression ofCSD2up-regulate ROS accumulation upon *M. oryzae* infection.

Fig. S6 Phylogenetic tree of SOD family members in rice.

Fig. S7 Overexpressing MIM398 or overexpression of miR398b target genes up-regulate CSD accumulation and enzyme activity upon *M. oryzae* infection.

Fig. S8 Overexpressing MIM398 or overexpression of miR398b target genes does not up-regulate total SOD enzyme activity upon *M. oryzae* infection.

Fig. S9 Overexpressing miR398b or mutations intarget genes does not affect CAT enzyme activity.

Table S1. Primers Used in This Study

Table S2. Guide RNAs and Target Sites used in CRISPR/ Cas9 technology

Table S3. Quantitative analysis of *M. oryzae* growth at the indicated time points

Methods S1H_2O_2measurement

H_2O_2 accumulation and cell death in infected leaves were observed following the procedure published previously (Xiao *et al.*, 2003). For observing *M. oryzae*-triggered H_2O_2 accumulation, leaves of six-leaf-stage rice seedlings were spray-inoculated with Guy11 (1×10^5 mL^{-1} spores) or water (mock). After 48 hr, DAB (3,3'-diaminobenzidine, Sigma, D8001)and trypan blue were used to stain H_2O_2 and cell death, respectively. In brief, 2.5-cm-long leafsections were placed in 1 mg/ ml DAB and incubated for 8 hat illumination. The DAB-stained leaves were double stained with trypan blueand observed with a microscope (Zeiss AxioImager A2).

References:

Xiao S, Brown S, Patrick E, Brearley C, Turner JG. 2003. Enhanced transcription of the arabidopsis disease resistance genes rpw8.1 and rpw8.2 via a salicylic acid-dependent amplification circuit is required for hypersensitive cell death. *Plant Cell* **15**: 33-45.

Fig. S1 Mutation sites of miR398b target genes in mutant lines. (a, c, e, g, i, k and m) Alignment of the sequences of guide RNAs with the genomic sequences of indicated mutants. The red bases indicate the deleted or substituted bases in mutants. (b, d, f, h, j, l and n) The alignment of protein sequences between mutants and WT. The yellow background show the identical amino acids between mutants and WT. the green background indicate the similar amino acids.

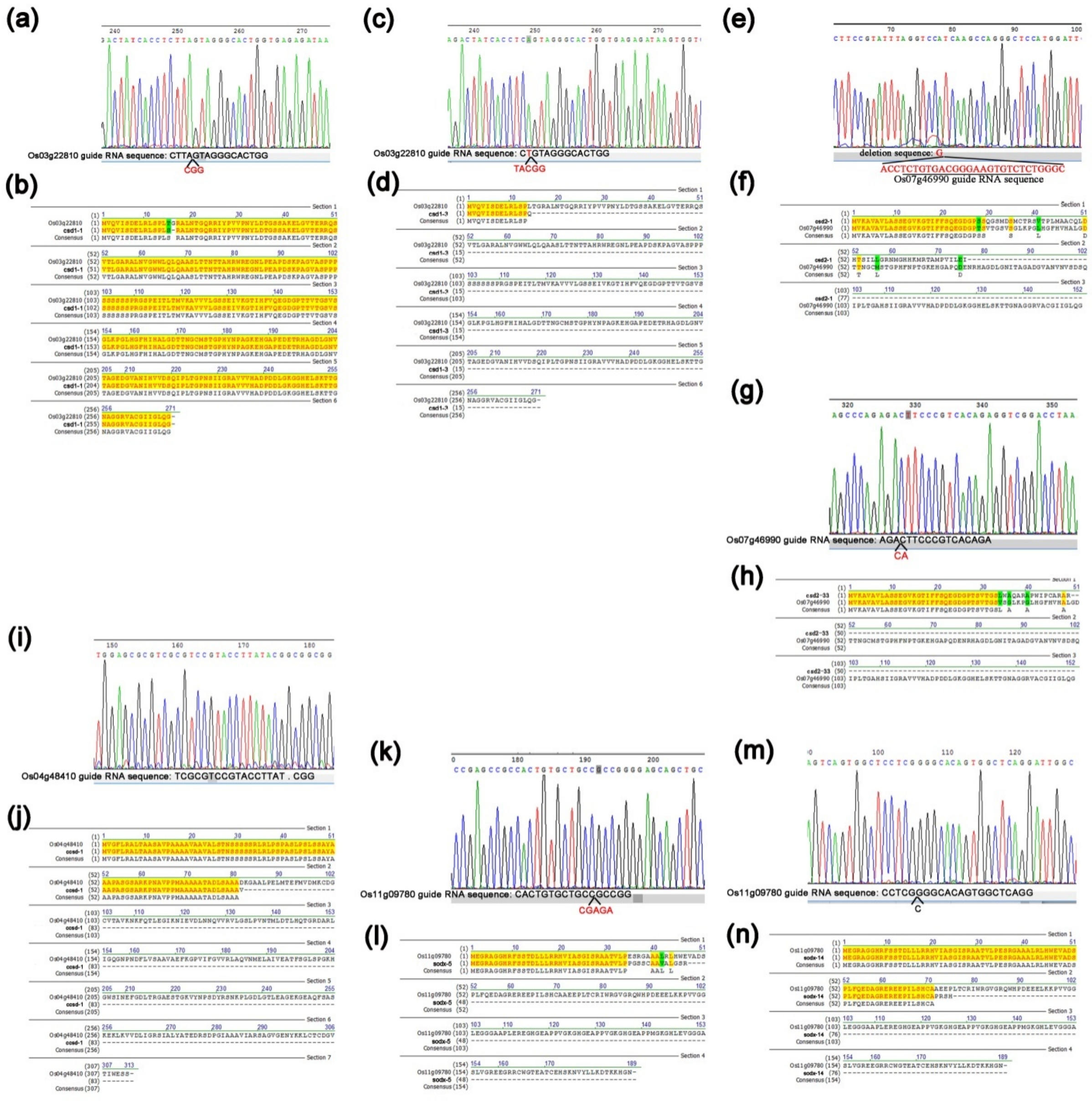

Fig. S1

Fig. S2 Overexpressing miR398b or mutations in target genes alters sensitivity to *M. oryzae*. (a) Representative leaf sections to show the blast disease phenotypes after spray-inoculated with *M. oryzae* strains 089 at 5 dpi. (b) The relative fungal mass on the inoculated leaves of the indicated lines. The relative fungal mass was measured by using the DNA level of *M. oryzaePot2* against the rice genomic ubiquitin DNA level. Values are means of three replications. Error bars indicate SD. Different letters above the bars indicate significant differences at P < 0.01 as determined by a one-way ANOVA followed by post hoc Tukey HSD analysis. The experiments were repeated two times with similar results.

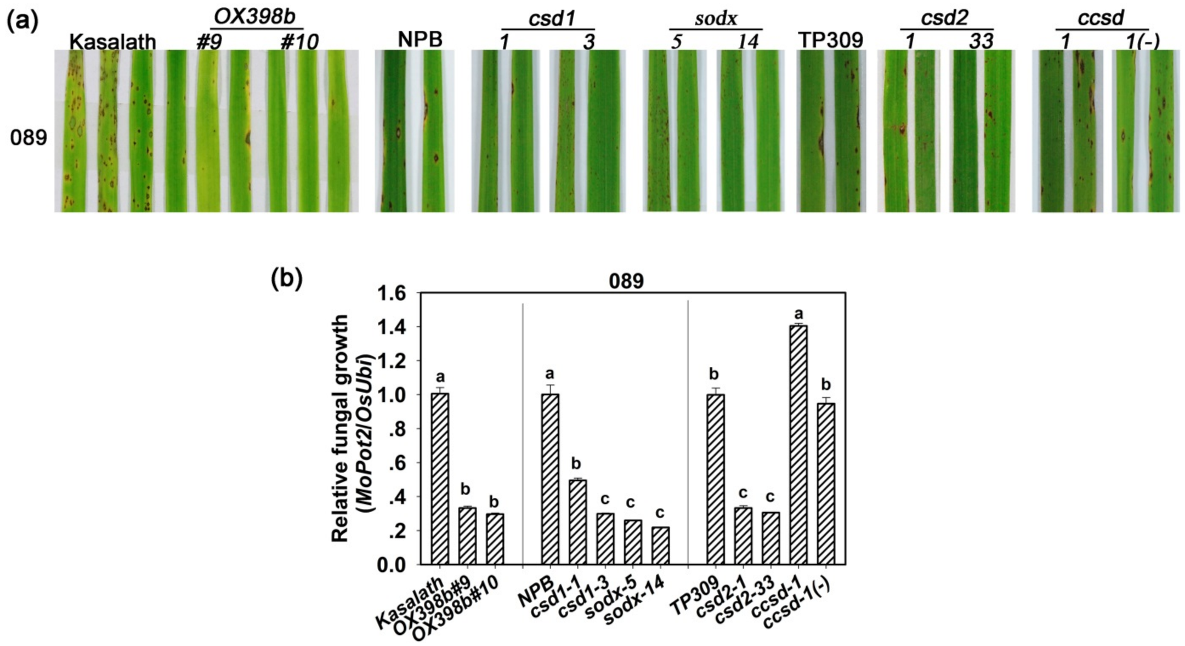

Fig. S2

Fig. S3 The mRNA and protein levels of CSD2 and CCSD increases in overexpressing transgenic lines. (a, b) Constructs of miR398b-insensitive versions $CSD2_m$-GFP and $CCSD_m$-GFP. The red letters indicate the mutations introduced into the miR398b target site in CCSD. (c, e) The RNA level of $CCSD_m$(c) and $CSD2_m$ (e) in WT and overexpressing transgenic lines. Ubiquitin served as the inter reference gene, and mRNA level was normalized to that in WT plants. Values are means of three replications. Error bars indicate SD. Different letters above the bars indicate significant differences at $P < 0.01$ as determined by a one-way ANOVA followed by post hoc Tukey HSD analysis. (d, f) Representative confocal images showing the subcellular accumulation of $CCSD_m$-GFP (d) and $CSD2_m$-GFP (f) in the indicated transgenic lines. The sheath cells of transgenic plants were observed to acquire images under laser scanning confocal microscopy (LSCM). Size bar, 10 μm.

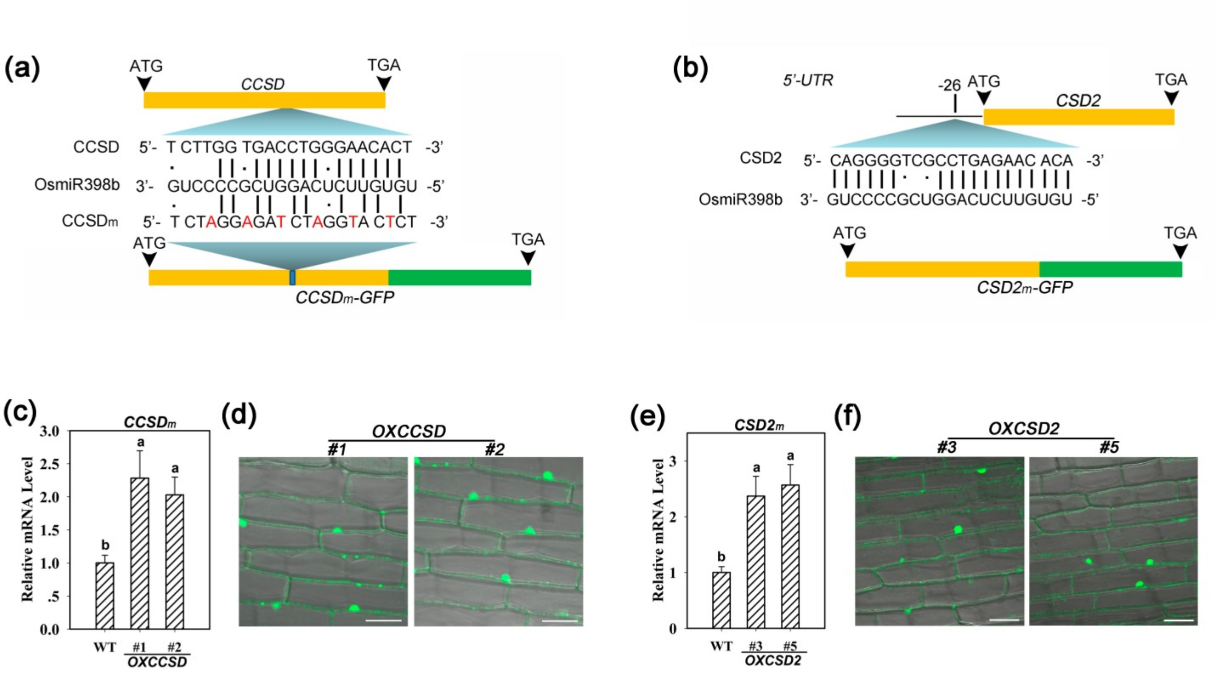

Fig. S3

Fig. S4 Representative leaf sections from the *OX398b* and mutant lines show the accumulation of H_2O_2 and $O{\cdot}_2^-$. The accumulation of H_2O_2 and $O{\cdot}_2^-$ were detected by DAB and NBT staining in the indicated treatment, respectively. The photo was captured from samples at 48 hpi with the *M. oryzae* strain Guy11; water was used as mock inoculation.

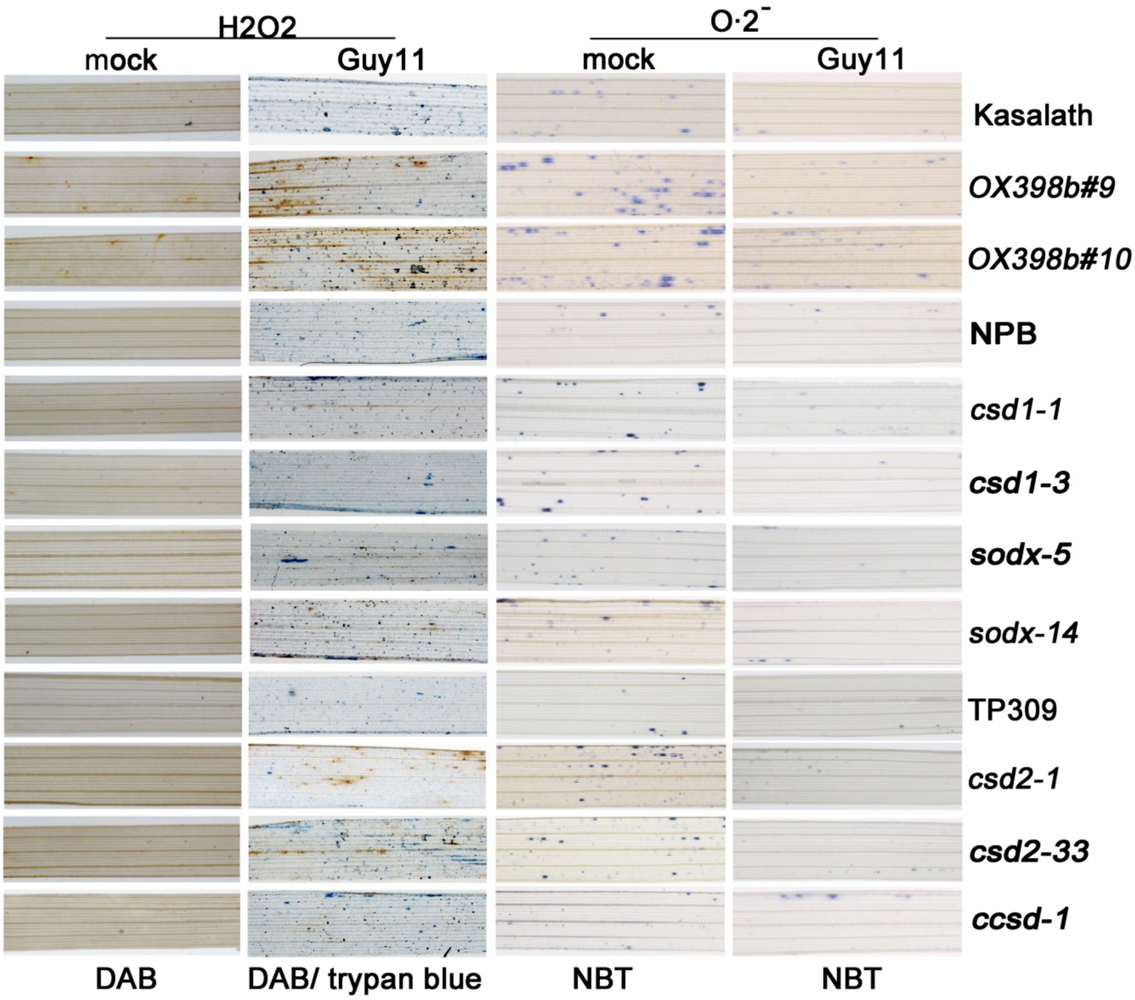

Fig. S4

Fig. S5 Overexpressing MIM398 or overexpression ofCSD2does not up-regulate ROS accumulation upon *M. oryzae* infection. (a) Representative leaf sections from the indicatedlines show the accumulation of H_2O_2 and $O{\cdot}_2^-$ detected by DAB and NBT staining in the indicated treatment. The photo was captured from samples at 48 hpi with the *M. oryzae* strain Guy11; water was used as mock inoculation. (b, c) Quantification of H_2O_2 (b) and $O{\cdot}_2^-$ (c) levels in leaves of the indicated lines with Guy11/mock treatment at 48 hpi, respectively. Values are means of three replications. Error bars indicate SD. Different letters above the bars indicate significant differences at $P < 0.01$ as determined by a one-way ANOVA followed by post hoc Tukey HSD analysis.

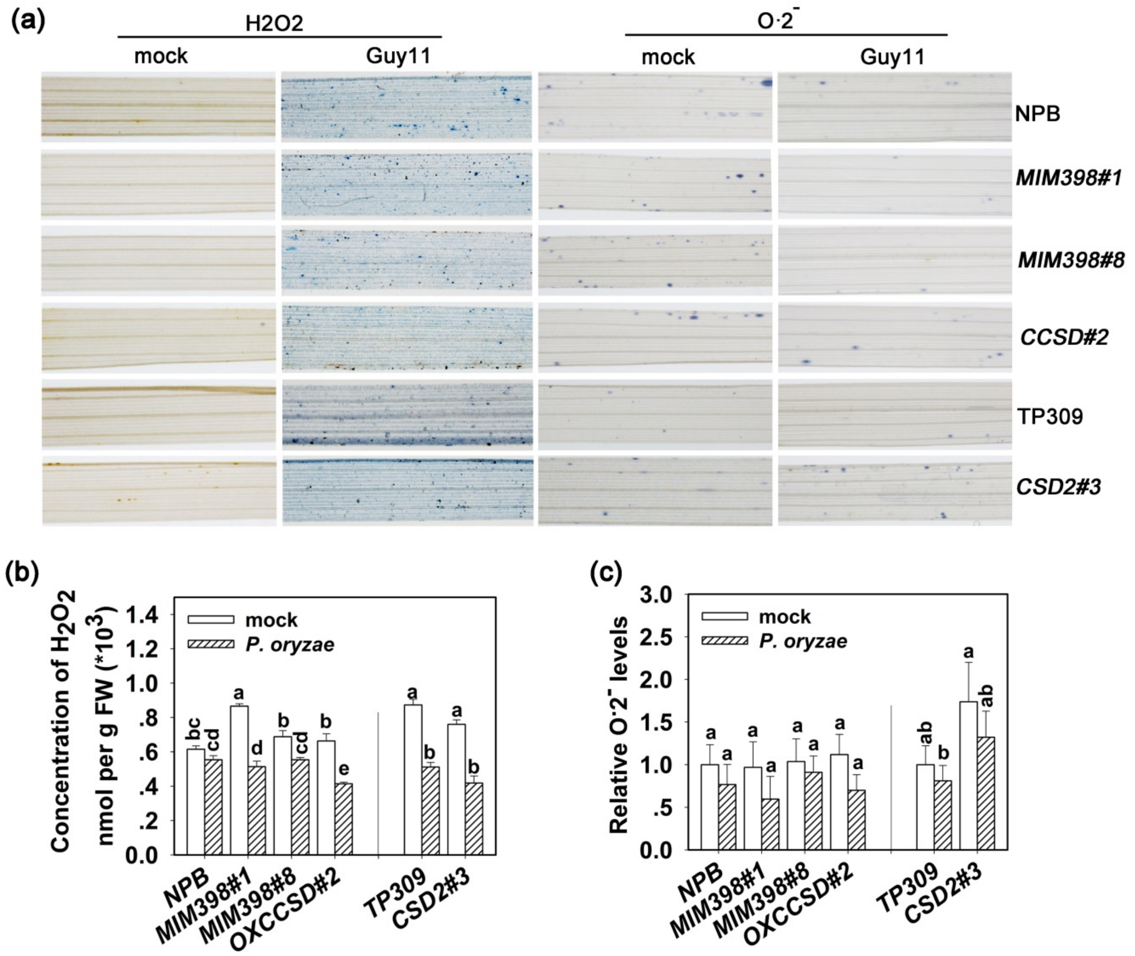

Fig.S5

Fig. S6 Phylogenetic tree of SOD family members in rice.

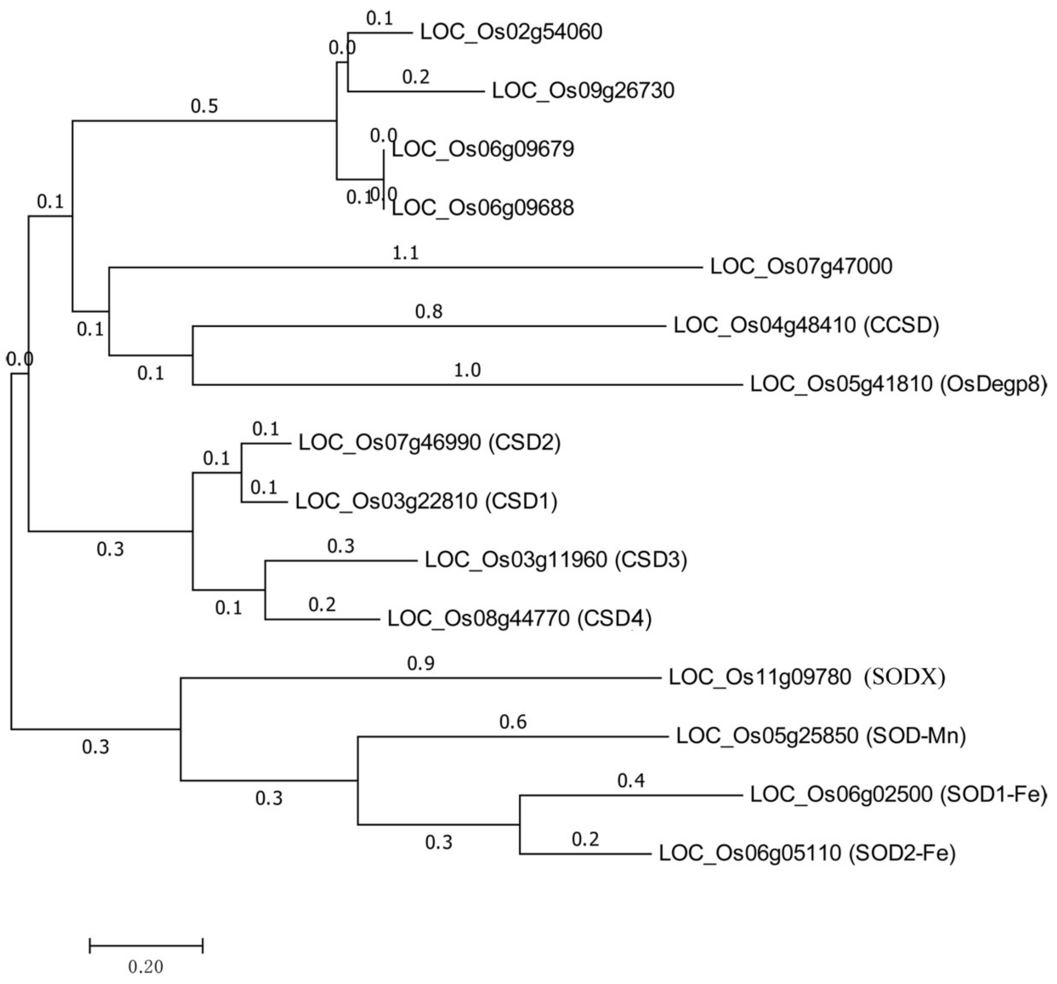

Fig. S6

Fig. S7 Overexpressing MIM398 or overexpression of miR398b target genes up-regulate CSD accumulation and enzyme activity upon *M. oryzae* infection. (a-d) Quantitative RT-PCR data show the expression pattern of the indicated genes in WT (NPB and TP309) and transgenic lines with Guy11/mock infection at 48 hpi. Relative mRNA level was normalized to that in WT mock samples. (e) The enzyme activity of CSD in transgenic lines and WT plants with Guy11/mock treatment. All results are the means of three replicates. Error bars indicate standard deviations. Different letters above the bars indicate significant differences at $P < 0.01$ as determined by a one-way ANOVA followed by post hoc Tukey HSD analysis. All the experiments were repeated two times with similar results.

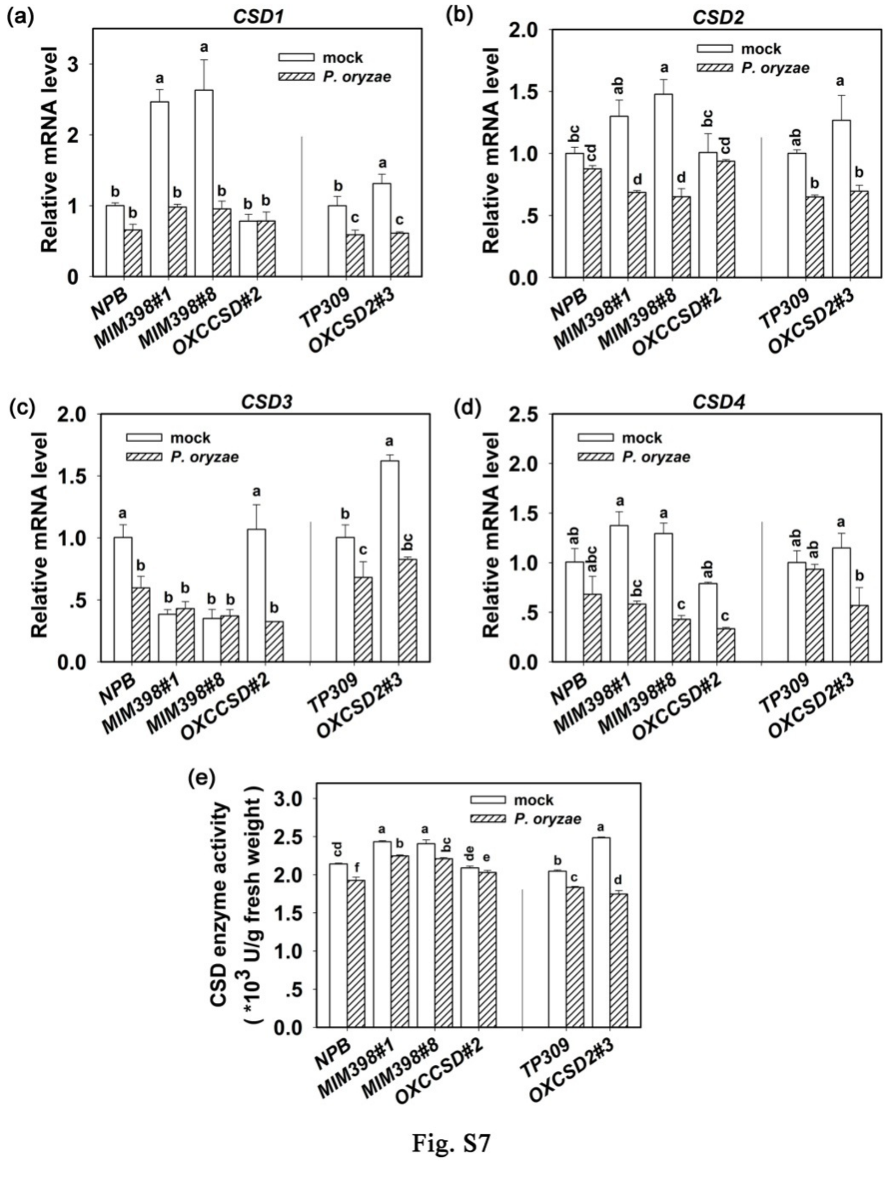

Fig. S7

Fig. S8 Overexpressing MIM398 or overexpression of miR398b target genes does not up-regulate total SOD enzyme activity upon *M. oryzae* infection. (a-d) Quantitative RT-PCR data show the expression pattern of the indicated genes in WT (NPB and TP309) and transgenic lines with Guy11/mock infection at 48 hpi. Relative mRNA level was normalized to that in WT mock samples. (e) The enzyme activity of SOD in transgenic lines and WT plants with Guy11/mock treatment. All results are the means of three replicates. Error bars indicate standard deviations. Different letters above the bars indicate significant differences at $P < 0.01$ as determined by a one-way ANOVA followed by post hoc Tukey HSD analysis. All the experiments were repeated two times with similar results.

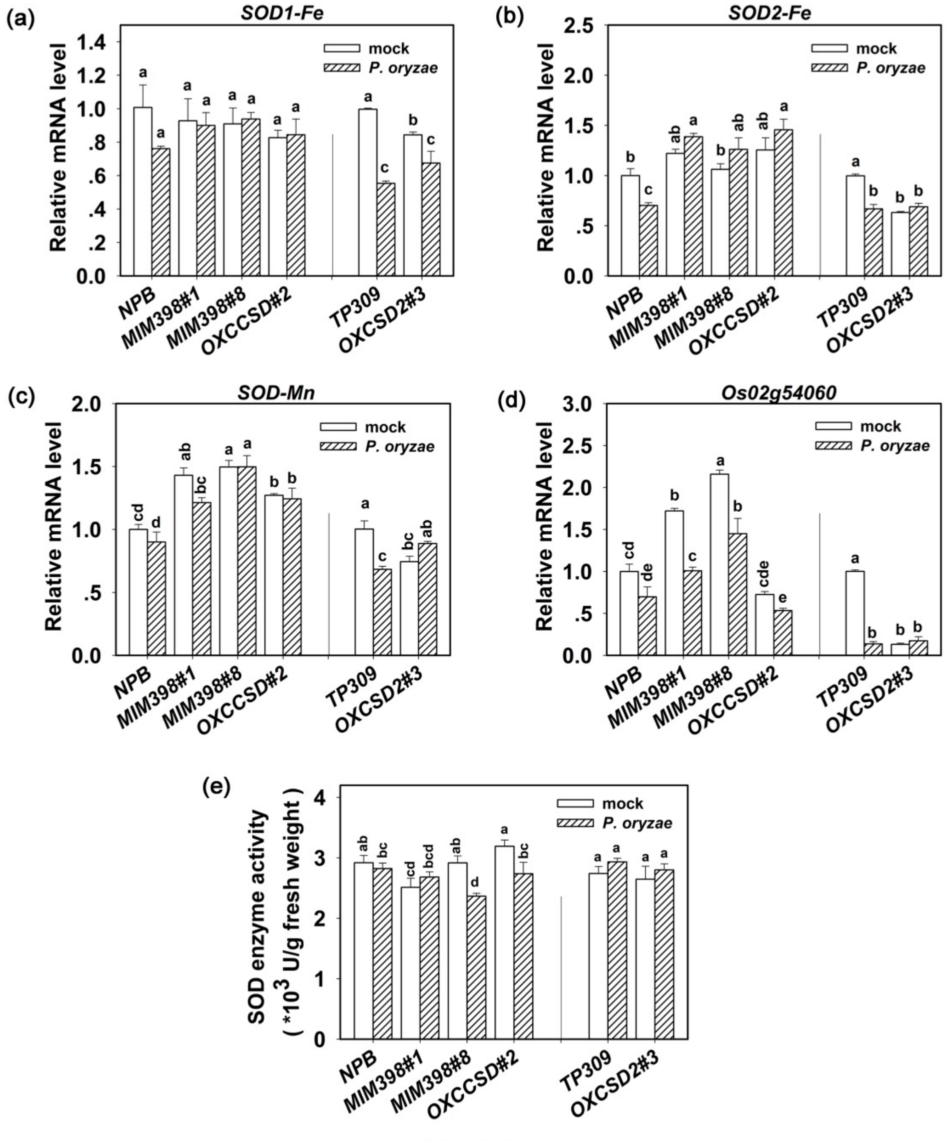

Fig. S8

Fig. S9 Overexpressing miR398b or mutations intarget genes does not affect CAT enzyme activity. The enzyme activity of CAT in transgenic lines and WT (Kasalath, NPB and TP309) plants with Guy11/mock treatment. Results are the means of four replicates. Error bars indicate standard deviations (SD). Different letters above the bars indicate significant differences at P < 0.01 as determined by a one-way ANOVA followed by post hoc Tukey HSD analysis. The experiments were repeated two times with similar results.

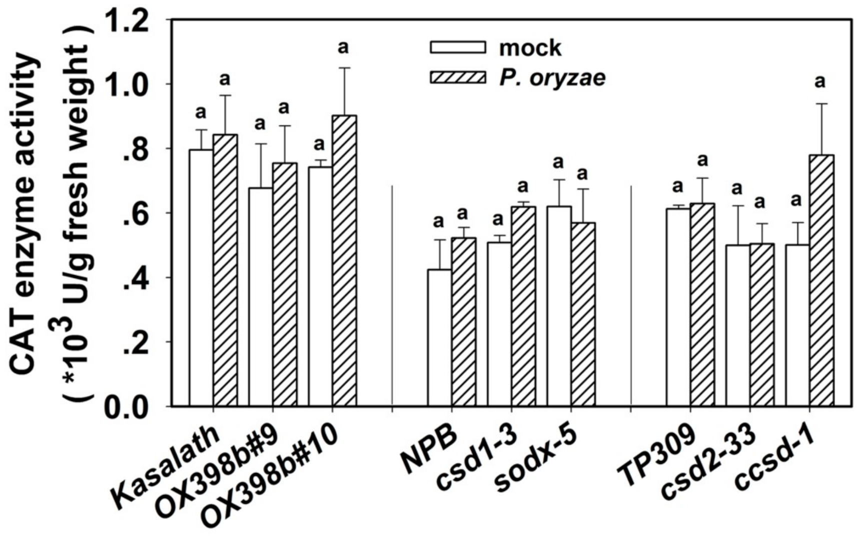

Fig. S9

Table S1. Primers Used in This Study

CCSD-KpnI-F	acaGGTACCgcacaaATGGTGGGCTTCCTG
CCSD-SpeI-R	ACCACTAGTGCTTGACTCCCAAATGGTGAC
CCSDm-F	TCTAGGAGATCTAGGTACTCTAGAAGCTGGAGAGAAGGGTG
CCSDm-R	TTCTAGAGTACCTAGATCTCCTAGAGGCTTATTGGATCTATAATC
CSD2-KpnI-F	ACAGGTACCATGGTGAAGGCTGTTGCTGTG
CSD2-SpeI-R	ACCACTAGTCTAACCCTGGAGTCCGATGAT
MIM398b-KpnI-F	ACAGGTACCAGCAGCAGGTTAGCTAAGCAC
MIM398b-SpeI-R	ACCACTAGTTTGAGTGGGGAGGCTTTGAG
CSD1-F for RT	ACCAATGGTTGCATGTCAAC
CSD1-R for RT	TGAATTTGGTCCAGTAAGTGG
CCSD-F for RT	TCACCTGGTAAACACGGATG
CCSD-R for RT	TGTTCCCAGGTCACCAAGAG
CSD2-F for RT	AAGAGGAGAGGGTGGGCAAC
CSD2-R for RT	AGTTGACATGCAGCCATTAGTGG
CSD3-F for RT	TGGGCGACCTGGGAAACATAG
CSD3-R for RT	GAGTTCATGACCACCCCTTCCT
CSD4-F for RT	TCCGTGTGACGGGACTTAC
CSD4-R for RT	GCCTCAGCTACACCTTCAGCAT
*SOD*X-F for RT	AGAGGGAAGGGCATGGAGAG
SODX-R for RT	TCCTCTCTCCCCACCAACGAC
SOD-Mn-F for RT	GAAGATGAGTGCAGAAGGTGC
SOD-Mn-R for RT	TTCTTGTACTGCAGGTAGTACGC
SOD1-Fe-F for RT	TGAGGATCGGAGATCTGACTATG
SOD1-Fe-R for RT	ATCCCCATTGACCTGCTGAG
SOD2-Fe-F for RT	TGCCATCAGTCCACTTGCAC
SOD2-Fe-R for RT	CATGCGTAGAGTGACAGTATCCC
Os02g54060-F for RT	AACTCCTGGTGGCCTATTGC
Os02g54060-R for RT	AGAGGACAGCCATTAGATCTGAC
*Rboh*D-F for RT	GGAGCGAGCTGATGAGTACA
*Rboh*D-R for RT	CAGCAGCTTCAGATGGTGAC
*Rboh*F-F for RT	GCTACACAAGCCAGGCACTA
*Rboh*F-R for RT	ACAGTGCAAGTACCCACAGG
*Rboh*B-F for RT	CAGCAAGGCGAAGAAGAAAC
*Rboh*B-R for RT	CCTCTGAACCACTCAAACGA
*MoPot*2-F for RT	ACGACCCGTCTTTACTTATTTGG
*MoPot*2-R for RT	AAGTAGCGTTGGTTTTGTTGGAT
OsUbi-F for RT	GCCCAAGAAGAAGATCAAGAAC
OsUbi-R for RT	AGATAACAACGGAAGCATAAAAGTC
csd2-F cas9	CGAGAACAGCTATCAAAATTCCCA
csd2-R cas9	TCTCCCAAGAGGGAGATGGT

ccsd-F cas9	ACAAATGGTGGGCTTCCTCC
ccsd-R cas9	CTAGCAGCATCGCAGCAAAC
sodx-F cas9	CCATTTTTCGTCCGTTCGCA
sodx-R cas9	CATGCCCTTCCCTCTCCAG
csd1-F cas9	AGTAGGACCATCCCATCCCC
csd1-R cas9	CGCATTGTGCTACCTGTCAC
$SODX_{ts}$-F	CATGCGCTGCTGGGGGACAGAGGCGACCTGTGAACACTCAAAGAACGTGTATGGTAC
$SODX_{ts}$-R	CATACACGTTCTTTGAGTGTTCACAGGTCGCCTCTGTCCCCCAGCAGCGCATGGTAC
$SODX_{mts}$-F	CATGCGCTGCTGGGGGACTGAAGCTACATGCGAGCATTCAAAGAACGTGTATGGTAC
$SODX_{mts}$-R	CATACACGTTCTTTGAATGCTCGCATGTAGCTTCAGTCCCCCAGCAGCGCATGGTAC

Table S2. Guide RNAs and Target Sites used in CRISPR/ Cas9 technology

	guide RNA	target site
**CSD1 gRNA-1**	GGTCCGTGACCTTGGGCGCA	AGTCCGTGACCTTGGGCGCAAGG
**CSD1 gRNA-2**	GCCAGTGCCCTACCCGTAAG	ACCAGTGCCCTACCCGTAAGAGG
**CSD2 gRNA-1**	GCACTTCAATCCTACTGGGA	ACACTTCAATCCTACTGGGAAGG
**CSD2 gRNA-2**	GCTGTGACGGGAAGTGTCTC	TCTGTGACGGGAAGTGTCTCTGG
**CCSD gRNA-1**	GCGCGTCCGTACCTTATCGG	TCGCGTCCGTACCTTATCGGCGG
**CCSD gRNA-2**	GGAGGGGAGCCGGAGTCTGG	CGAGGGGAGCCGGAGTCTGGAGG
**SODX gRNA-1**	GACTGTGCTGCCCGAGAGCC	CACTGTGCTGCCCGAGAGCCGGG
**SODX gRNA-2**	GCTGAGCCACTGTGCCGCCG	CCTGAGCCACTGTGCCGCCGAGG

Table S3. Quantitative analysis of *M. oryzae* growth at the indicated time points

Time points	Lines	Not germinated conidia	Germinated conidia	Invasive hypha in local cell	Invasive hypha in neighbour cells
24 hpi	NPB	0.25±0.43	5.81±2.29	93.94±2.68	0.00±0.00
	MIM398#1	0.23±0.40	5.91±0.91	92.03±1.63	1.83±0.53*
	MIM398#8	0.60±1.04	6.33±1.21	91.77±0.94	1.30±0.35*
48 hpi	NPB	0.00±0.00	0.38±0.66	12.94±1.74	86.67±2.40
	MIM398#1	0.00±0.00	0.36±0.62	5.43±0.90*	94.22±1.52*
	MIM398#8	0.00±0.00	0.34±0.59	5.17±2.65*	94.49±3.23*
24 hpi	TP309	3.34±1.49	93.49±2.09	3.17±2.79	0.00±0.00
	OXCSD2#3	3.52±1.54	91.68±1.47	4.80±1.08	0.00±0.00
48 hpi	TP309	1.24±0.98	8.61±2.33	43.32±1.54	46.83±1.55
	OXCSD2#3	0.40±0.69	14.34±1.59*	18.94±1.54**	66.32±2.34**
24 hpi	NPB	0.25±0.43	5.81±2.29	93.94±2.68	0.00±0.00
	OXCCSD#2	0.00±0.00	24.90±4.86**	75.10±4.86**	0.00±0.00
48 hpi	NPB	0.00±0.00	0.38±0.66	12.94±1.74	86.67±2.40
	OXCCSD#2	0.00±0.00	0.49±0.85	14.90±4.45	91.27±7.56
24 hpi	NPB	0.25±0.43	5.81±2.29	93.94±2.68	0.00±0.00
	csd1-3	3.18±0.51	9.56±1.06	87.26±1.15*	0.00±0.00
	sodx-5	1.91±0.64	10.01±3.76	88.08±3.12*	0.00±0.00
48 hpi	NPB	0.00±0.00	0.38±0.66	12.94±1.74	86.67±2.40
	csd1-3	0.00±0.00	0.81±1.41	24.17±5.68*	75.02±4.46*
	sodx-5	0.00±0.00	0.93±1.23	27.17±3.69*	72.02±2.41*
24 hpi	TP309	1.17±1.26	23.92±5.68	74.92±4.47	0.00±0.00
	csd2-33	4.37±0.69	20.14±0.49	75.48±1.18	0.00±0.00
	ccsd-1	0.72±1.25	5.67±1.15*	93.61±0.14*	0.00±0.00
48 hpi	Top309	0.00±0.00	0.69±1.20	16.87±3.81	82.43±2.62
	csd2-33	0.00±0.00	1.18±2.04	28.65±4.36*	70.17±3.04*
	ccsd-1	0.00±0.00	0.46±0.79	10.24±2.84	88.90±3.41*

Notes: *indicate significant differences at P < 0.05 and ** indicate significant differences at P < 0.01 as determined by a one-way ANOVA followed by post hoc Tukey HSD analysis. Error values indicate standard deviations (SD).
